# The proapoptotic gene interferon regulatory factor-1 mediates the antiproliferative outcome of paired box 2 gene and tamoxifen

**DOI:** 10.1038/s41388-020-01435-4

**Published:** 2020-08-25

**Authors:** Shixiong Wang, Venkata S. Somisetty, Baoyan Bai, Igor Chernukhin, Henri Niskanen, Minna U. Kaikkonen, Meritxell Bellet, Jason S. Carroll, Antoni Hurtado

**Affiliations:** 1 Cell Cycle Regulations Group, Nordic EMBL Partnership, Centre for Molecular Medicine Norway (NCMM), University of Oslo, Blindern, P.O. 1137, 0318 Oslo, Norway; 2 Cancer Research UK Cambridge Institute, University of Cambridge, Robinson Way, Cambridge, CB2 0RE UK; 3 A.I. Virtanen Institute for Molecular Sciences, University of Eastern Finland, PO Box 1627, 70211 Kuopio, Finland; 4 Vall Hebron Institute of Oncology, Barcelona, Spain; 5 Vall Hebron University Hospital, Barcelona, Spain; 6 Cancer Genomics and Proteomics Group, Department of Biomedical Sciences, University of Barcelona, Casanova, 143, 08014 Barcelona, Spain; 7 August Pi i Sunyer Research Center (IDIBAPS), c/Rosselló, 149-153, 08036 Barcelona, Spain

**Keywords:** Breast cancer, Prognostic markers

## Abstract

Tamoxifen is the most prescribed selective estrogen receptor (ER) modulator in patients with ER-positive breast cancers. Tamoxifen requires the transcription factor paired box 2 protein (PAX2) to repress the transcription of ERBB2/HER2. Now, we identified that PAX2 inhibits cell growth of ER+/HER2− tumor cells in a dose-dependent manner. Moreover, we have identified that cell growth inhibition can be achieved by expressing moderate levels of PAX2 in combination with tamoxifen treatment. Global run-on sequencing of cells overexpressing PAX2, when coupled with PAX2 ChIP-seq, identified common targets regulated by both PAX2 and tamoxifen. The data revealed that PAX2 can inhibit estrogen-induced gene transcription and this effect is enhanced by tamoxifen, suggesting that they converge on repression of the same targets. Moreover, PAX2 and tamoxifen have an additive effect and both induce coding genes and enhancer RNAs (eRNAs). PAX2–tamoxifen upregulated genes are also enriched with PAX2 eRNAs. The enrichment of eRNAs is associated with the highest expression of genes that positivity regulate apoptotic processes. In luminal tumors, the expression of a subset of these proapoptotic genes predicts good outcome and their expression are significantly reduced in tumors of patients with relapse to tamoxifen treatment. Mechanistically, PAX2 and tamoxifen coexert an antitumoral effect by maintaining high levels of transcription of tumor suppressors that promote cell death. The apoptotic effect is mediated in large part by the gene interferon regulatory factor 1. Altogether, we conclude that PAX2 contributes to better clinical outcome in tamoxifen treated ER-positive breast cancer patients by repressing estrogen signaling and inducing cell death related pathways.

## Introduction

Breast cancer is the most common malignancy in women and overall is the second most common cancer worldwide, with more than 2 million new cases diagnosed globally each year. Breast cancer is also a heterogenous disease at both histological and molecular levels. According to gene expression patterns in tumors, breast cancer is classified into several intrinsic molecular subtypes, and each subtype differs in the expression of key molecular markers and prognosis [1]. Despite the complexity, around 70% of tumors are composed of the estrogen receptor (ER) α-positive luminal subtype. Endocrine treatments that target estrogen signaling are the main strategies in the adjuvant setting and their use in the neoadjuvant scenario is gaining progressive interest, particularly in the postmenopausal population. For decades, the selective ER modulator tamoxifen (Tam) has been the mainstay of the adjuvant treatment for premenopausal women with breast cancer and it is still widely used in this context. Tam is a chemical compound that antagonizes estrogen action by competing with estrogen for binding to ER. By contrast, the use of aromatase inhibitors (AIs) is the preferred adjuvant treatment in postmenopausal women. AIs inhibit the synthesis of estradiol and then prevent the activation of ER. Moreover, Tam is the treatment of choice in postmenopausal women who do not tolerate an AI [2].

Previously, we and others reported that paired box 2 protein (PAX2) is a key factor in breast cancer by repressing the transcription of ERBB2/HER2 in ER-positive (ER+) breast cancer [3, 4]. Moreover, it has been described that high level of PAX2 is associated with better survival in Tam treated ER+ breast cancer patients [3, 5]. However, it is not clear how PAX2 can modulate ER activity in ER+ and HER2-negative (HER2−) tumors. PAX2 is a protein that belongs to the paired box transcription factor family with DNA binding domains represented by paired domain. PAX2 plays an important role in tissue development, such as the renal tissue morphogenesis [6], and progesterone-dependent mammary growth. PAX2 can either activate or repress gene transcription by recruiting regulatory proteins such as PTIP [7] or GRG4 [8, 9] through protein interaction with the C-terminal transactivation domain. PTIP or GRG4 further recruit enzymes that catalyze histone modifications to activate or repress transcription, respectively [7, 10].

In this study, we have characterized the role of PAX2 in ER+/HER2− breast cancer cells, specifically under conditions where ER has been inhibited with an antagonist. We have used Global Run-On sequencing (GRO-seq) analysis to reveal substantial transcriptional alterations in cells expressing different levels of PAX2. Moreover, we show that increased PAX2 expression improves Tam response by two means. Firstly, PAX2 represses the transcription of estrogen-induced genes in an additive manner with Tam treatment leading to a cell growth inhibition. Secondly, PAX2 induces both coding gene transcripts and enhancer RNAs (eRNAs) nearby genes enriched at cell death processes. PAX2 ChIP-seq revealed that Tam enhances the binding of PAX2 toward the promoter regions of proapoptotic genes with PAX2-induced eRNAs, contributing to increased gene expression. The high expression of three of these genes predicts survival in luminal tumors and the expression of them is significantly reduced in tumors with relapse to Tam. One key target gene is interferon regulatory factor-1 (IRF1), which is a proapoptotic factor. The expression of PAX2 and its target gene IRF1 is essential for Tam-induced apoptosis.

## Results

### The levels of PAX2 expression determine the benefit of Tam to inhibit cell growth

**Fig. 1:**
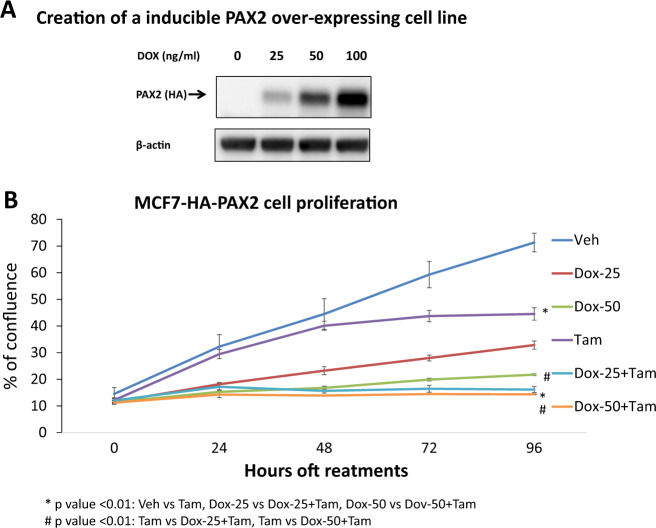
PAX2 expression in ER+/HER2− tumors is associated with response to tamoxifen. **a** Protein blot of PAX2-HA and beta actin (as loading control) of stable MCF-7-breast cancer cell inducible for PAX2-HA expression. The expression of PAX2-HA is induced by doxycycline and different concentrations. **b** Cell growth assay of stable MCF-7-breast cancer cell inducible for PAX2-HA expression. Doxycycline and non-doxycycline treated cells (25 or 50 ng/ml) were treated with vehicle (control) or tamoxifen (1 μM) for 96 h. Tamoxifen was added every 24 h.

The role of PAX2 regulating the transcription of ERBB2/HER2 in breast cancer was previously reported in ER+ cells treated with Tam [4, 5]. The repression of ERBB2 expression by ER-Tam was shown to require PAX2. Moreover, clinical studies have observed a significant association between high expression of PAX2 and better disease-free survival in patients treated with Tam [5]. Now, we aimed to investigate how increased levels of PAX2 expression improved Tam action in ER+/HER2− cell line. We used MCF-7 cells, which express moderate levels of PAX2 as reported previously [4]. We created an inducible PAX2 overexpressing MCF-7 cell line and tested the expression of the ectopic PAX2 on cell viability. MCF-7 PAX2 overexpressing cells were plated in estrogen-rich, full culture media and treated with Dox to induce the ectopic overexpression of PAX2. After 16 h of Dox treatment at different concentrations, PAX2 levels were increased in a Dox dependent manner (Fig. 1a). Next, we investigated how PAX2 overexpression influenced the basal growth of cells and whether Tam treatment affected this. Cells were treated with three concentrations of Dox (0, 25, and 50 ng/ml) to induce different amounts of PAX2 expression and subsequently treated with vehicle (Veh) or Tam. The degree of cell growth inhibition correlated with PAX2 expression levels (Fig. 1b), confirming the previous data implicating PAX2 as a repressor in breast cancer [5]. Moreover, the treatment with Tam resulted in greater cell growth inhibition with intermediate levels of PAX2 expression, suggesting that Tam can potentiate the antiproliferative effects of patients with moderate expression of PAX2. Altogether, these findings support the hypothesis that high PAX2 expression inhibits cell growth and PAX2 expression improves the efficacy of Tam in ER+/HER2− cancer cells.

### PAX2–Tam regulates the transcription of gene transcripts related to cell growth and death

**Fig. 2:**
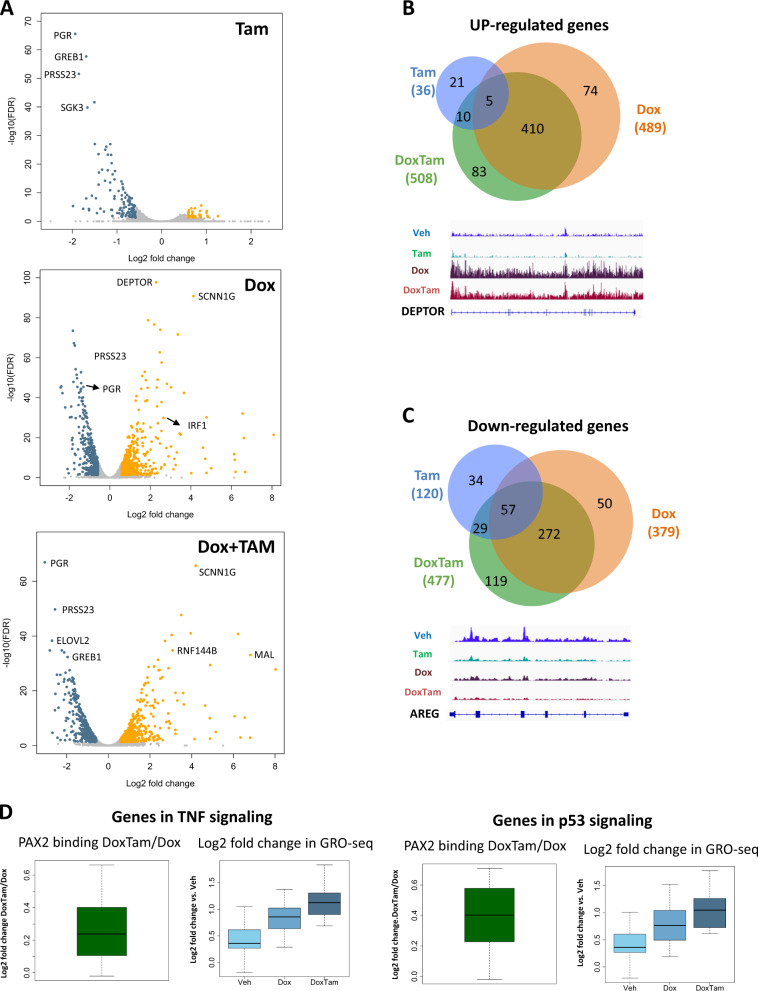
PAX2 regulates a gene signature in MCF-7 cells. **a** Volcano plots of genes regulated in different treatments (tamoxifen, PAX2, and PAX2–tamoxifen) detected in GRO-seq experiment. **b**, **c** upper panel: Venn diagrams showing the overlap of up or downregulated genes detected in GRO-seq between different treatments respectively. **b**, **c** lower panel: genome browser examples illustrating different patterns of regulation of PAX2 up or downregulated genes. **d** Box plots of sequencing data for PAX2-HA ChIP-sequencing (left) and gene expression (right) of PAX2-regulated genes with PAX2-HA binding sites at their TSS. Genes enriched at TNF (top) and p53 (bottom) signaling are indicated. The ChIP-sequencing data are indicated as fold change of PAX2 binding of tamoxifen (DoxTam) vs non-tamoxifen (Dox). The expression data are indicated as log2 fold change of cells overexpressing PAX2 (dox) or overexpressing PAX2 and tamoxifen treatment (DoxTam) relative to non-PAX2 overexpressing cells (Veh).

In order to investigate the precise role of PAX2 and Tam in ER+/HER2− tumors, we first determined the expression of coding genes and eRNAs regulated by PAX2, Tam, or the combination of both. MCF-7 PAX2 overexpressing cells were plated in full culture media and treated with Dox (50 ng/ml Dox) to induce high expression of PAX2. After 16 h of Dox treatment, 1 µM Tam was added to control or Dox treated cells for 6 h. In order to determine both coding genes and eRNAs, we performed GRO-seq for each treatment (Veh, Tam, Dox (PAX2), and Tam + Dox (PAX2)) with two biological replicates. Libraries were prepared and sent for high throughput sequencing, with a minimum of 14 million aligned reads. First, differentially expressed coding genes were called as described in the methods and only genes above 1.5-fold change (treatments vs control cells) and FDR < 0.05 were considered significantly regulated. In total, 1054 differentially expressed genes were identified in at least one of the three treatments (Fig. 2a): 156 genes in Tam treatment (36 up and 120 down), 868 genes in PAX2 overexpression (489 up and 379 down), and 985 in Tam treated cells overexpressing PAX2 (508 up and 477 down). The overlap of up- and downregulated genes between different treatments is shown in Fig. 2b, c. Cells overexpressing PAX2 were largely unaffected by cotreatment with Tam (Fig. 2b). However, a subset of genes (90 genes) was significantly upregulated in conditions of high PAX2 expression and Tam treatment (Fig. 2c). In cells without PAX2 overexpression, we identified a very limited overlap with Tam-induced genes but the majority of Tam-repressed genes (62.6%) were also downregulated in PAX2 overexpressing cells. Examples of genes regulated only by PAX2 or regulated by both PAX2 and Tam are shown at the bottom of Fig. 2b, c.

In order to characterize the biological effect of PAX2 and Tam regulated transcriptome, pathway enrichment analysis of differentially regulated genes detected by GRO-seq was performed. As predicted, significant enrichment in estrogen response signaling was observed in genes downregulated by Tam or PAX2 (Supplementary Fig. 1A, B), providing support that PAX2 elicits its antiproliferative effects in ER+ cancer cells, via modulation of ER target genes. Moreover, when analyzing genes downregulated by both Tam and PAX2, estrogen signaling was the most enriched pathway, with transmembrane receptor protein tyrosine kinase signaling pathway also enriched (Supplementary Figs. 1C and 2). When focusing specifically on genes induced by PAX2 expression or Tam as single treatments, we observed enrichment for two cytokine-related pathways (TNFα signaling via NFκB and interferon alpha response) in PAX2 but not in Tam group of genes (Supplementary Fig. 3A, B). The most enriched pathways observed in PAX2 overexpressing cells with Tam were hallmark of TNFα signaling and p53 (Supplementary Fig. 3C), suggesting that PAX2 and Tam might induce transcription of tumor suppressors.

Next, we aimed to investigate whether the changes in transcription due to Tam treatment might be directly regulated by PAX2. For that, we performed chromatin immunoprecipitation (ChIP)-sequencing of PAX2 in MCF-7 cells overexpressing PAX2 and treated with Tam. We had assessed numerous endogenous PAX2 antibodies for ChIP-seq but none could be validated. As such, we exploited the PAX2 overexpressing system, since PAX2 was tagged with hemagglutinin (HA). As control, we used cells without PAX2 overexpression. For that, MCF-7 PAX2 overexpressing cells were plated in full culture media and treated with 50 ng/ml of Dox to induce the ectopic overexpression of PAX2. After PAX2 overexpression, 1 µM Tam was added to the cells for 6 h and two biological replicates (Supplementary Fig. 4) of ChIP-seq were conducted as described in “Methods.” We identified a total of 84,730 PAX2 peaks in PAX2 overexpressing cells and 90,577 peaks in PAX2 overexpressing cells treated with Tam (Supplementary Fig. 5A, B). Almost all the PAX2 binding events for non-Tam treated cells where shared with the peaks found in cells treated with Tam (92%). Moreover, we observed a modest increase of PAX2 binding in Tam treated cells (14% of peaks were only observed in PAX2 expressing cells treated with Tam). We investigated what fraction of PAX2-regulated genes had PAX2 binding events nearby (±1.5 kb) their transcription start sites (TSS). Interestingly, we found that more than 60% of the PAX2 and Tam upregulated genes had at least one PAX2 binding site adjacent to their TSS (Supplementary Fig. 5C). Conversely, we found a moderate fraction (36%) of PAX2 downregulated genes with PAX2 sites (Supplementary Fig. 5C). In support of the enrichment of p53 and TNFα signaling pathways, we observed that PAX2 binding in cells treated with Tam was significantly increased around TSS of genes enriched within those pathways (Fig. 2d). Importantly, the impact of Tam on PAX2 binding correlated positively with the expression of genes enriched in TNFα and p53 pathways (Fig. 2d and Supplementary Fig. 5D) but not to non-PAX2-regulated genes (Supplementary Fig. 6). Altogether, our findings supported the hypothesis that PAX2 has a dual effect: it is able to repress the transcription of genes in estrogen response and to activate transcription of genes related to cell death and growth arrest. Furthermore, the PAX2 effect was enhanced by simultaneous Tam administration (Supplementary Figs. 7 and 8).

### PAX2–Tam induce transcripts with clinical outcome

**Fig. 3:**
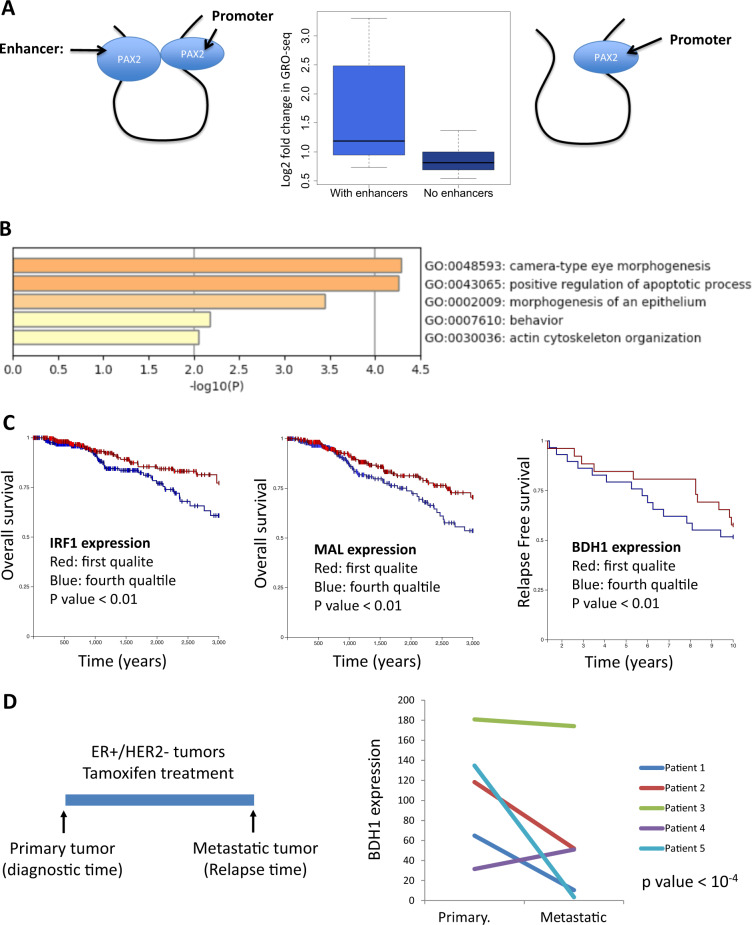
PAX2 upregulated genes with eRNAs predict tamoxifen outcome. **a** Boxplot indicating the expression of PAX2 upregulated genes with (*n* = 16) and without (*n* = 79) PAX2 eRNAs. **b** List of enriched pathways in PAX2 upregulated genes with PAX2 eRNAs. **c** Kaplan–Meier curves of genes with significant overall survival (IRF1 and MAL) and relapse-free survival (BDH1). The analysis was performed by means of using public clinical databases available at UCSC Xena (browser: https://xenabrowser.net/heatmap/). **d** Paired samples (primary and metastatic tumors) of patients initially diagnosticated with ER+/HER2− tumors, which were treated with tamoxifen for 2 years and stopped responding to treatment. The expression by NanoString of BDH1 was investigated in both samples and in each of the five patients.

Our findings have suggested that PAX2 induces the expression of proapoptotic genes by interacting to the promoters of their target genes. Moreover, it is well known that gene transcription is regulated by the expression of eRNAs, which are located mainly at intergenic regions. Hence, we hypothesized that PAX2 might be binding to nearby enhancer regions to control the transcription of eRNAs. For that we determined whether PAX2, Tam, or the combination of both might impact the expression of eRNAs at intergenic regions. The differentially expressed eRNAs at intergenic regions were called as described in “Methods” and only eRNAs with 1.5-fold change (treatments vs control cells) and FDR < 0.05 were considered significantly regulated. In total, 620 differentially expressed eRNAs were identified in at least one of the three treatments (Supplementary Fig. 9): 77 in Tam treatment (9 up and 68 down), 518 in PAX2 overexpression (173 up and 245 down), and 507 in Tam treated cells overexpressing PAX2 (209 up and 298 down). Next, we determined how many of the PAX2-regulated genes were associated with PAX2 eRNAs. Our findings revealed that 16 PAX2 upregulated genes were associated with PAX2-induced eRNAs. Then, we compared the expression of the upregulated genes with or without PAX2-induced eRNAs. The results revealed that PAX2 upregulated genes with eRNAs were significantly upregulated compared to the upregulated genes without eRNAs (Fig. 3a). Importantly, those genes were also enriched in positive regulation of apoptosis (Fig. 3b). Based on our findings, we hypothesized that the expression of that subset of PAX2 highly expressed genes might prevent breast cancer tumors to relapse on Tam treatment. To validate our hypothesis, we determined whether the expression of these genes (Fig. 3c) might have an impact in patient survival and metastases in luminal breast cancer patients. Our analysis revealed that overexpression of 2 out of these 16 genes (IRF1 and MAL) was associated with longer survival. In addition, we identified that the overexpression of one of those genes (BDH1) was associated with a significant relapse-free survival (RFS). Next, we determined the expression of BDH1 in paired tumor samples (primary and metastatic) from patients who initially responded but develop resistance to Tam. Our analysis indicated that the expression of BDH1 gene was significantly downregulated in metastatic tumors when compared to the primary tumors in four out of five patients investigated (Fig. 3d). The high expression of BDH1 induces autophagy [11], which is considered a preliminary step to apoptosis. Overall, our findings were supporting the idea that PAX2–Tam induces massive transcription of genes that promotes cell death in breast cancer patients and that the loss of their expression is associated with resistance to Tam.

### PAX2 and Tam-induced apoptosis is mediated by IRF1

**Fig. 4:**
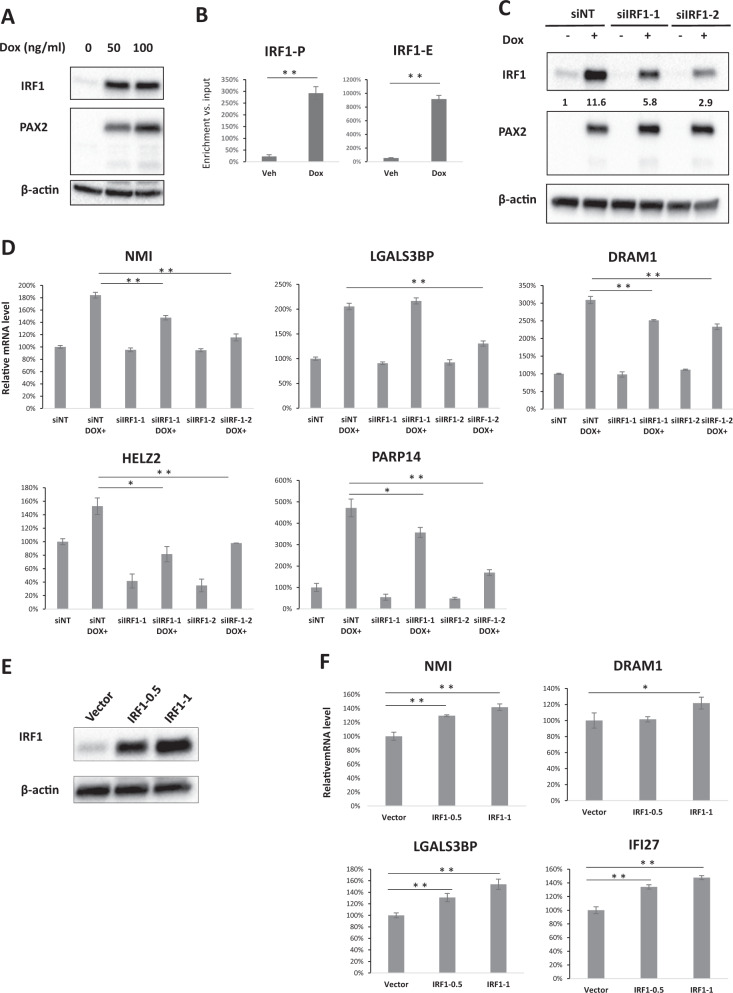
PAX2-induced transcription is partially dependent on IRF1. **a** Western blotting showing IRF1 induction by PAX2 overexpression. MCF-7-PAX2 cells were treated with Dox for 16 h, IRF1, PAX2, and β-actin levels were determined with western blotting. **b** PAX2 (HA antibody) ChIP experiment with MCF-7-PAX2 cells treated with vehicle (Veh) and 50 ng/ml doxycycline (Dox). Two sites located at IRF1 promoter (IRF1-P) or IRF1 enhancer (IRF1-E) were tested with real-time PCR (*n* = 3, *p < 0.05, **p < 0.01). Pull-down DNA amount was normalized to the input DNA of each sample, and data are presented as the percentage of input with ±s.d. as the error bar. **c** Western blotting of MCF-7-PAX2 cells transfected with nontargeting siRNA (siNT), and two siRNAs targeting IRF1 (siIRF1-1 and siIRF1-2). MCF-7-PAX2 cells were transfected with siNT, or siIRF1. IRF1, PAX2, and β-actin level were detected by Western blotting. IRF1 protein level was quantified by normalization against beta actin level. **d** Real-time PCR analysis of genes upregulated by PAX2. MCF-7-PAX2 cells were transfected with nontargeting siRNA (siNT) or IRF1 siRNA followed by doxycycline treatment. mRNA levels of genes were measured with real-time PCR, and result was normalized first to UBC and then to the average of Veh sample. The data are represented as the mean of independent replicates ± s.d. (*n* = 3, *p < 0.05, **p < 0.01). **e** MCF-7 cells were transfected with pCI-neo empty vector or with increasing amount of pCI-neo-IRF1 vector. Forty-eight hours after transfection, IRF1 and β-actin level was determined with western blotting. **f** MCF-7 cells were transfected with pCI-neo empty vector or with increasing amount of pCI-neo-IRF1 vector, then mRNA levels of different genes were detected with real-time PCR, result was normalized first to UBC and then to the average of Veh, and data are presented as the percentage of input with ±s.d. as the error bar (*n* = 3, *p < 0.05, **p < 0.01).

Next, we aimed to investigate the putative role of PAX2 and Tam in regulating apoptosis. Therefore, we explored the PAX2 upregulated genes with eRNAs, and among them we identified that IRF1, a tumor suppressor gene, was one of the top PAX2-induced genes. We validated that IRF1 protein level was significantly upregulated by PAX2 overexpression (Fig. 4a). PAX2 ChIP followed by real-time PCR confirmed PAX2 binding at both the promoter and enhancer of the IRF1 gene (Fig. 4b), indicating direct transcriptional regulation. Considering that IRF1 is an important transcription factor mediating apoptosis in breast cancer, we hypothesized that the induction of genes related to growth arrest and cell death by PAX2 could be partially attributed to IRF1 upregulation. In order to assess this hypothesis, two different siRNAs against IRF1 were validated, with differing degrees of silencing (Fig. 4c). The dependence of IRF1 on PAX2-induced transcription was assessed by real-time PCR in MCF-7-PAX2 cells transfected with nontargeting siRNA or siRNAs targeting IRF1 (Fig. 4d and Supplementary Fig. 10). To complement the IRF1 knockdown work, IRF1 was ectopically overexpressed in wild-type MCF-7 cell line (Fig. 4e, f). Transcription of IRF1 dependent genes was activated by IRF1 overexpression in MCF-7 cells (Fig. 4f). Altogether, our findings show that the transcription of a subset of PAX2–Tam upregulated genes is dependent of IRF1.

**Fig. 5:**
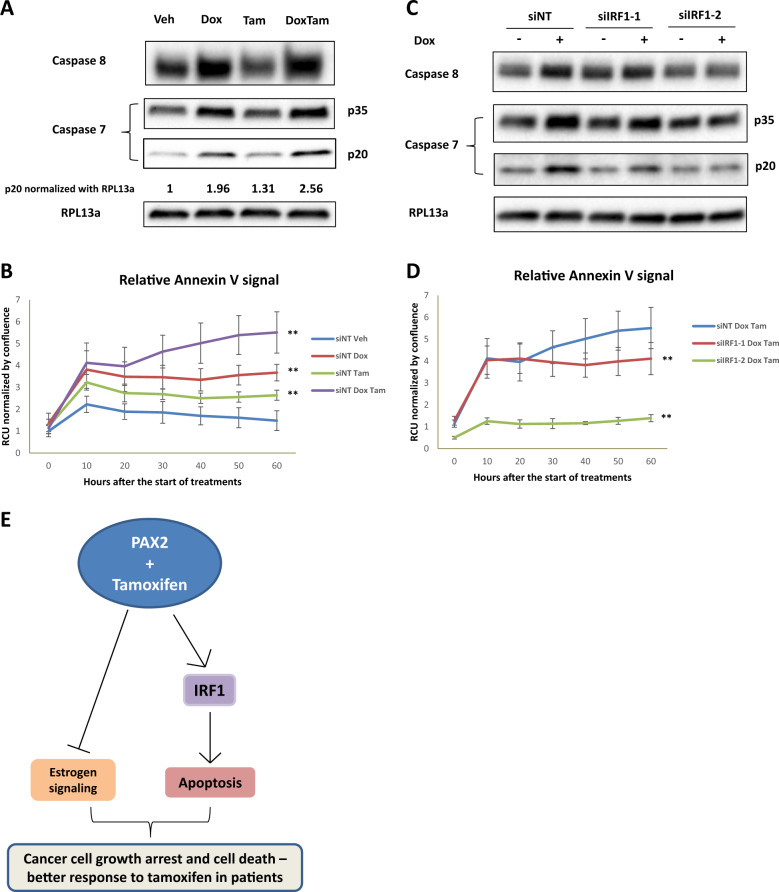
PAX2 induces apoptosis via IRF1. **a** Western blotting showing induction of caspase 7 and 8 level by PAX2. MCF-7-PAX2 cells were treated with vehicle (Veh), 50 ng/ml doxycycline (Dox), 1 μM tamoxifen (Tam) or the combination of both for 48 h, then whole cell lysate was prepared and analyzed by using western blotting with caspase 7, caspase 8, and RPL13a antibodies, RPL13a is used as loading control. Cleaved caspase 7 was quantified normalization against RPL13a. **b** MCF-7-PAX2 cells were transfected with siNT or siIRF1 for 24 h, then treated with or without 50 ng/ml doxycycline (Dox) for 48 h. Whole cell lysate was prepared and analyzed with western blotting with caspase 7, caspase 8, and RPL13a antibodies. **c**, **d** Real-time Annexin V assay showing PS externalization. MCF-7-PAX2 cells were transfected with siNT, or siIRF1 followed by treatments with vehicle (Veh), 50 ng/ml doxycycline (Dox), 1 μM tamoxifen (Tam), or the combination of both. PS externalization was measured according to the methods session. The data are represented as the mean of independent replicates ± s.d. (*n* = 5). **e** The model of actions of PAX2 in ER-positive breast cancer cells.

Given the results that some of the PAX2 genes are regulated by IRF1 and the expression of IRF1 predicts survival in breast cancer patients, we examined whether PAX2 was able to induce apoptosis through IRF1 in MCF-7 cells. IRF1 can induce caspase and we therefore assessed the status of caspase 8 and 7 with PAX2 overexpression. The level of pro-caspase 8, 7, and activated cleaved caspase 7 increased when PAX2 was overexpressed, compared with the nontreated control (Fig. 5a). Moreover, Tam enhanced PAX2-induced apoptosis shown as increased cleaved caspase 7 (Fig. 5a), suggesting that the combination of PAX2 overexpression and Tam could induce higher level of apoptosis in MCF-7 cells. The finding was further validated by real-time Annexin V staining assay, which showed increased Annexin V signal in the combination of PAX2 overexpression and Tam compared with either individual treatment (Fig. 5b and Supplementary Fig. 11). Then, by using IRF1 siRNAs we aimed to validate the role of IRF1 in PAX2 mediated apoptosis. The knock down of IRF1 could revert the induction of caspases 7 and 8 and cleaved caspase 7 (Fig. 5c), and furthermore the Annexin V signal induced by the combination of PAX2 overexpression and Tam (Fig. 5d and Supplementary Fig. 11). Based on the results above, we conclude that PAX2 is able to induce apoptosis in MCF-7 cells and that is enhanced by Tam treatment by upregulating proapoptotic genes such as IRF1 (Fig. 5e). Considering the heterogeneous nature of breast tumor, a few key experiments were repeated in another ER+/HER2 low cell line ZR-75-1. Results showed that PAX2 could inhibit cell proliferation and induce higher level of apoptosis (Annexin V assay) together with Tam (Supplementary Fig. 12), confirming the findings that discovered in MCF-7 as described above.

## Discussion

The results of this work have demonstrated that PAX2 induces the transcription of genes key for the initiation of apoptosis in ER+/HER2− breast cancer cells. This effect is mediated, in part, by the transcription factor IRF1, which is directly upregulated by PAX2. IRF1 is an important factor mediating the response to anti-estrogens in ER+ breast cancer cells [12]. In fact, the downregulation of IRF1 protects cells from PAX2-induced growth inhibition and apoptosis. At molecular level, IRF1 leads to the initiation of apoptosis by activating cell cycle inhibitors such as p21cip1 or p27kip1, caspases (CASP1, CASP3, CASP7, CASP8), and/or Fas ligand [13]. In addition, IRF1 could also exert its function by facilitating p53 acetylation, which can stabilize the protein [14, 15]. In agreement with this idea, we demonstrated that p53 protein level was increased by PAX2, whereas the mRNA level remained unaffected (Supplementary Fig. 8G, H). This concept is consistent with the fact that PAX2 and its gene target IRF1 have an antitumor effect by promoting apoptosis [12]. In addition, we have discovered that the PAX2 apoptotic effect is enhanced by the anti-estrogen drug Tam. Tam enhances the expression of some PAX2-induced genes key in the control of p53 function. For instance, Tam enhances the upregulation of RNF114B, which functions as an E3 ubiquitin ligase for Pirh228 [16]. RNF144B binds and ubiquitinates Pirh2 for proteasomal degradation, which is negative regulator of p53. These findings support the idea that the expression of PAX2-induced genes in luminal tumors might function as a marker of good prognosis and also as a predictive factor of benefit from Tam. Breast cancer patients with luminal tumors expressing PAX2 might be responding better to Tam in comparison to patients with undetectable PAX2 expression. The expression of PAX2 has a double effect in luminal tumors: it induces transcription of proapoptotic genes and represses the transcription of estrogen-induced genes key to cell division. Therefore, by inhibiting the cell division and also by inducing apoptosis PAX2 and Tam might prevent the tumor growth and ultimately lead to more efficient Tam response.

The results of this study suggest that the expression of PAX2 is associated to a better prognosis in patients with luminal tumors. Moreover, our findings suggest that patients with increased PAX2 protein levels might show better outcomes for Tam treatment. In agreement with our findings, our clinical data support that ER+/PAX2+/HER2− patients without Tam treatment (or any endocrine treatment) have shorter RFS compared to patients treated with Tam (median RFS 5.3 years in Tam vs 4 years in nontreated patients; data not shown). In both the neoadjuvant and adjuvant scenarios, Tam has shown to be slightly inferior to AI both in the postmenopausal population with early breast cancer in the premenopausal patients on GnRH analogs [2]. One possible explanation of the poorer results of Tam could be due to the fact that only 40–60% of these luminal patients are positive for the expression of PAX2 [3, 17], which might be specifically predictive of Tam response. If that was the case, it is reasonable to think that Tam might provide similar benefit than AI in a well selected population for PAX2 positivity, and the neoadjuvant scenario would be the most appropriate platform to test this hypothesis. Regardless of that, AI are associated to a non-meaningless toxicity (arthromyalgies, asthenia, sexual dysfunction among others) leading to endocrine therapy discontinuation in a substantial percentage of women, for whom Tam is still a suitable alternative. Whether the expression of PAX2 upregulated transcripts is a better indicative marker for Tam outcome than PAX2 itself in the neoadjuvant setting should be evaluated in the future.

### Sequencing data

The GEO accession numbers of the data are GSE139928 and GSE140060 for GRO-seq and ChIP-seq, respectively.

## Methods

### Cell culture

ER+ breast cancer and HER2− cell lines MCF-7 and ZR-75-1 were purchased from American Type Culture Collection (Manassas, VA). MCF-7 is cultured in Dulbecco’s Modified Eagle Medium (41966-052, Gibco) supplemented with 10% of fetal bovine serum (10500-064, Gibco), and ZR-75-1 is cultured in RPMI-1640 medium (61870010, Gibco) supplemented with 10% FBS. MCF-7-PAX2 stable cell line is kept in additional 200 μg/ml of hygromycin B and 5 μg/ml puromycin.

### Plasmids and cloning construction

pLV.ExSi.P/Hygro-CMV-TET3G and pLV.ExSi.P/Puro-TRE3G-PAX2/HA vectors were purchased from Cyagen (Santa Clara, CA, USA). pCI-Neo mammalian overexpression vector was purchased from Promega (E1841). IRF1 full length sequence was amplified from MCF-7 cDNA with corresponding primers. IRF1 cDNA and PAX2-HA sequence were cloned into pCI-neo with NheI and MluI restriction sites.

### Transfection

MCF-7-PAX2-HA cells were transfected with siRNAs targeting IRF1 (Thermo Fisher, AM16708, siRNA ID: 115266 (siIRF1-1) and 115267 (siIRF1-2)), siControl Non-targeting (siNT) (SI03650318 from Promega) using Lipofectamine RNAiMax (Life technologies) to a final concentration of 20 nM following the reverse transfection protocol from the manufacturer in full culture media.

For transient transfection with pCI-neo-IRF1 or PAX2 vector, MCF-7 and ZR-75-1 cells were plated into a six-well plate around 70% confluences. Transfection was carried out with Lipofectamine 3000 (Invitrogen) following the manufacturer’s protocol.

### Lentivirus production and stable cell line generation

Lentiviral delivery plasmid pLV.ExSi.P/Hygro-CMV-TET3G and pLV.ExSi.P/Puro-TRE3G-PAX2/HA were cotransfected respectively with packaging plasmids into 293T cells plated in 10 cm dishes. Collection of supernatants was done twice at 48 and 72 h post transfection followed by concentration with Lenti-X concentrator (#631232, Clontech). In order to generate MCF-7-PAX2 stable cell line, MCF-7 cells were firstly infected with virus carrying pLV.ExSi.P/Hygro-CMV-TET3G and selected with 200 μg/ml hygromycin B for 20 days to get MCF-7-Tet3G cell line. Then, MCF-7-Tet3G cell line was infected with virus containing pLV.ExSi.P/Puro-TRE3G-PAX2/HA and selected with 200 μg/ml hygromycin B and 2 μg/ml puromycin for 20 additional days.

### Western blotting

Cells were lysed (50 mM Tris-HCl, pH 7.4; 100 mM NaCl; 1 mM EDTA; 1 mM EGTA; 1 mM NaF; 0.1% SDS; 0.5% sodium deoxycholate; 1% Triton-X-100; 2 mM sodium orthovanadate; Protease Inhibitor Cocktail (Thermo Fisher)). Protein lysate was resolved using precast SDS-PAGE gels, and transferred to PVDF membrane. Blots were blocked and incubated overnight at 4 °C with primary antibodies, followed by three washes with TBS with 0.1% Tween 20 (TBST). Then, membranes were incubated with HRP-conjugated second antibodies against corresponding species of primary antibodies for 1 h and followed by three washes with TBST. Finally, membranes were developed with SuperSignal™ West Pico (Thermo Fisher) or SuperSignal™ West Femto (Thermo Fisher). Primary antibodies used are as bellow: PAX2-HA tag (ab9110) antibodies were purchased from Abcam; IRF1 (8478S), caspase 7 (9492S), caspase 8 (4790s), RPL13a (2765S), and β-Actin (4970S) antibodies were from Cell Signalling Technology.

### Cell proliferation assay

Cell proliferation assay was performed with the Incucyte system (Essen BioScience). MCF-7-PAX2 cells were plated in a 24-well plate at 10% confluence (0.02 × 10^6^/well) in full culture media. On the first day after plating, cells were treated with 1 μM Tam (H7905, Sigma) and doxycycline (Dox). ZR-75-1 were transfected with pCI-neo empty vector or pCI-neo-PAX2-HA vector, which is followed by trypsinization and subplating into a 96-well plate 4–6 h after transfection. Cells were treated with Veh or 1 μM Tam in the following day. The proliferation (termed phase contrast in the Incucyte software) was monitored by the Incucyte system every 3 h for at least 96 h. For MCF-7-PAX2 and for ZR-75-1 were used three and four wells (technical replicates) for each treatment, respectively. The data were plotted showing the average of phase contrast with standard deviation as the error bar.

### RNA extraction and real-time PCR

MCF-7-PAX2 cells were plated into a six-well plate in full culture media with 5% of FBS with or without siRNA transfection (siNon-targeting or siIRF1). MCF-7 and ZR-75-1 cells were transfected with pCI-neo, pCI-neo-IRF1, or pCI-neo-PAX2-HA. Cells were used for RNA extraction and total RNA was isolated with TRIzol Reagent (15596018, Invitrogen) following the manufacturer’s protocol.

A total of 2 μg of RNA was used for cDNA synthesis with SuperScript III (18080093, Invitrogen), and real-time PCR was performed with Power SYBR Green PCR Master Mix (4368702, Applied Biosystems). Primers for genes tested are listed in Supplementary Materials.

Reference genes for normalization were selected following geNorm algorithm [18]. Eight reference genes (UBC, GADPH, RPL13A, SDHA, ACTB, B2M, HMBS, HPRT1) were tested in all cell lines and treatments, among them RPL13A and UBC were selected for MCF-7, and RPL13A and SDHA for ZR-75-1. In all real-time PCR experiments, three technical replicates were included for each sample, and two-tailed Student’s *t* test was done to determine the *p* value between samples.

### GRO-seq library preparation

Global run-on and library preparation for sequencing was performed as previously described [19] with minor modifications. Briefly, 2 × 10^6^ MCF-7-PAX2 cells were plated into each 15 cm plate in DMEM with 5% FBS. Three days after plating, cells were treated with Veh (control) or 50 ng/ml Dox to overexpress PAX2. Sixteen hours after Dox treatment, cells were treated with either Veh (control) or 1 μM 4-Hydroxytamoxifen (Sigma). Nuclei isolation was performed 6 h after stimulation and 5 × 10^6^ nuclei were used for each run-on reaction. Two biological replicates were produced for each treatment (control, Dox, Tam and Dox + Tam). Br-UTP was incorporated into on-going transcription by run-on reaction that was performed at 30° for 5 min. Total RNA was extracted with TRIzol Reagent (Life Technologies) and fragmented with RNA Fragmentation Reagent (Life Technologies). Fragmented RNA was purified with P-30 column (Bio-Rad, Hercules, CA, USA), which was followed by T4 polynucleotide kinase (New England Biolabs) treatment to dephosphorylate the 3′ end of RNA fragments. Br-UTP labeled RNA was enriched twice with anti-BrdU beads (Santa Cruz Biotechnology) and precipitated overnight. Poly(A) tailing was done using *E.coli* Poly(A) Polymerase (New England Biolabs), followed by reverse transcription with oNTI-223-index: /5Phos/GATCGTCGGACTGTAGAACTCTGAAC/iSp18/TCAGACGTGTGCTCTTCCGATCTTTTTTTTTTTTTTTTTTTTVN, which allows custom barcoding. Exonuclease I (New England Biolabs) was used to remove excess oligo after reverse transcription. DNA–RNA duplex was purified with ChIP DNA Clean & Concentrator Kit (Zymo Research Corporation) followed by RNAse H treatment. cDNA was circularized with CircLigase II (Epicentre) and amplified with oNTI-201: AATGATACGGCGACCACCGACAGGTTCAGAGTTCTACAGTCCGACG and oNTI-200:CAAGCAGAAGACGGCATACGAGATXXXXXXGTGACTGGAGTTCAGACGTGTGCTCTTCCGATCT (XXXXXX is barcode used for specific sample) for 12–14 cycles. Final PCR product was purified by running 10% TBE gel and cleaned up. Libraries were sequenced on Illumina Genome Analyzer HiSeq 2000 according to the manufacturer’s instructions.

### GRO-seq data processing and data analysis

GRO-seq data were first trimmed from the 3′ end to remove PolyA tail with Homer tools followed by quality filtering with FASTX Toolkit with the criteria that minimum 97% of base pairs should have quality scores higher than 10. Trimmed and quality-filtered data were aligned to human genome hg19 with Bowtie [20]. Differential expression analysis was performed with edgeR [21], genes with RPKM > 0.5 were considered expressed, 1.5-fold change with FDR < 0.05 was considered significant differential expression. Intergenic transcripts (eRNAs) were called by Homer software with a minimum length of 300 bp. Differentially expressed eRNAs were detected by following the same criteria to coding genes. Regulated eRNAs were annotated with Homer software according to the nearest gene. Regulated genes, which were the nearest genes of regulated eRNAs in the same treatment, were selected for further analysis.

### Chromatin immunoprecipitation

PAX2 genomic distribution was identified by using the cross-linking ChIP protocol as described previously [22]. A total of 2 × 10^6^ MCF-7-PAX2 cells were plated into each 15 cm plate in DMEM with 5% FBS 3 days after plating, cells were treated with Veh (control) or 50 ng/ml Dox to overexpress PAX2. Sixteen hours after Dox treatment, cells were treated with either Veh (control) or 1 μM 4-Hydroxytamoxifen for 6 h. Then, cells were fixed for 10 min with 1% formaldehyde and then quenched with glycine (125 mM). Chromatin was incubated overnight at 4 °C with Chip grade HA tag antibody (10 μg of ab9110) and equal amounts of Protein A&G Agarose Beads (Life Technologies). Immunoprecipitated genomic DNA was sent to Norwegian sequencing center for library preparation and sequenced with Illumina NextSeq 500.

### ChIP-seq data analysis

ChIP-seq raw data were aligned to human genome assembly hg19 with Bowtie. Then, PCR duplicates were removed with SAMtools [23], followed by peak calling with MACS2 [24] with *q* value cutoff 0.01. Correlation between replicates was analyzed with deepTools [25]. Overlap between datasets was analyzed with bedtools [26]. ER ChIP-seq data were adapted from previous study [27], and was processed as mentioned above.

### Pathway enrichment analysis

Pathway enrichment analysis was performed with Metascape (http://metascape.org) [28] and GSEAPreranked in Gene set enrichment analysis [29] with regulated gene lists derived from GRO-seq analysis.

### Analysis of mRNA expression in breast cancer sections

Total RNA (150 ng) was shipped to the NanoString nCounter^®^ Human mRNA Expression Assay analysis. RNA was incubated in the presence of mRNA specific probes. To account for minor differences in hybridization and purification efficiencies raw data were adjusted using a technical normalization factor calculated from six internal positive spike controls present in each reaction. Background hybridization was corrected by deducting the negative control mean plus two standard deviations calculated from eight negative controls.

### Annexin V assay

The Annexin V assay measuring the externalization of phosphatidylserine (PS) was carried out by using Incucyte Annexin V Red Reagent (4641, Essen BioScience). MCF-7-PAX2 cells were plated into a 96-well plate in full media with 5% FBS with or without transfection of siRNA (siNT or siIRF1). Twenty-four hours after plating cells were treated with Veh, 1 µM Tam, 50 ng/ml Dox, or the combination of both. At the same time the Incucyte Annexin V Red Reagent was added into the media in a 1:200 dilution. ZR-75-1 cells were first transfected with pCI-neo or pCI-neo-PAX2-HA, followed subplating into 96-well plate 4–6 h after transfection. Cells were treated with Veh or 1 µM Tam from the following day. The signal of Annexin V was measured every 2 h by quantifying total red object integrated intensity signal (total RCU), and the cell confluence was measured by phase contrast. Relative Annexin V signal was calculated by normalizing the RCU parameter against cell confluence.

## Supplementary information

Supplementary Figure Legend

Supplementary figures

Supplementary materials

## References

[1] Prat A, Perou CM (2011). Deconstructing the molecular portraits of breast cancer. Mol Oncol.

[2] Pistelli M, Mora AD, Ballatore Z, Berardi R (2018). Aromatase inhibitors in premenopausal women with breast cancer: the state of the art and future prospects. Curr Oncol.

[3] Hurtado A, Holmes KA, Geistlinger TR, Hutcheson IR, Nicholson RI, Brown M (2008). Regulation of ERBB2 by oestrogen receptor-PAX2 determines response to tamoxifen. Nature.

[4] Beauchemin D, Lacombe C, Van Themsche C (2011). PAX2 is activated by estradiol in breast cancer cells of the luminal subgroup selectively, to confer a low invasive phenotype. Mol Cancer.

[5] Jahangiri R, Mosaffa F, Gharib M, Emami Razavi AN, Abdirad A, Jamialahmadi K (2018). PAX2 expression is correlated with better survival in tamoxifen-treated breast carcinoma patients. Tissue Cell.

[6] Sharma R, Sanchez-Ferras O, Bouchard M (2015). Pax genes in renal development, disease and regeneration. Semin Cell Dev Biol.

[7] Patel SR, Kim D, Levitan I, Dressler GR (2007). The BRCT-domain containing protein PTIP links PAX2 to a histone H3, lysine 4 methyltransferase complex. Dev Cell.

[8] Cai Y, Brophy PD, Levitan I, Stifani S, Dressler GR (2003). Groucho suppresses Pax2 transactivation by inhibition of JNK-mediated phosphorylation. EMBO J.

[9] Eberhard D, Jimenez G, Heavey B, Busslinger M (2000). Transcriptional repression by Pax5 (BSAP) through interaction with corepressors of the Groucho family. EMBO J.

[10] Daniel JA, Santos MA, Wang Z, Zang C, Schwab KR, Jankovic M (2010). PTIP promotes chromatin changes critical for immunoglobulin class switch recombination. Science.

[11] Martinez-Outschoorn UE, Lin Z, Whitaker-Menezes D, Howell A, Sotgia F, Lisanti MP (2012). Ketone body utilization drives tumor growth and metastasis. Cell Cycle.

[12] Schwartz JL, Shajahan AN, Clarke R (2011). The role of interferon regulatory factor-1 (IRF1) in overcoming antiestrogen resistance in the treatment of breast cancer. Int J Breast Cancer.

[13] Dai C, Krantz SB (1999). Interferon gamma induces upregulation and activation of caspases 1, 3, and 8 to produce apoptosis in human erythroid progenitor cells. Blood.

[14] Dornan D, Eckert M, Wallace M, Shimizu H, Ramsay E, Hupp TR (2004). Interferon regulatory factor 1 binding to p300 stimulates DNA-dependent acetylation of p53. Mol Cell Biol.

[15] Ito A, Lai CH, Zhao X, Saito S, Hamilton MH, Appella E (2001). p300/CBP-mediated p53 acetylation is commonly induced by p53-activating agents and inhibited by MDM2. EMBO J.

[16] Sane S, Rezvani K. Essential roles of E3 ubiquitin ligases in p53 regulation. Int J Mol Sci. 2017;18. 10.3390/ijms18020442. 10.3390/ijms18020442 PMC5343976 28218667

[17] Silberstein GB, Dressler GR, Van Horn K (2002). Expression of the PAX2 oncogene in human breast cancer and its role in progesterone-dependent mammary growth. Oncogene.

[18] Vandesompele J, De Preter K, Pattyn F, Poppe B, Van Roy N, De Paepe A (2002). Accurate normalization of real-time quantitative RT-PCR data by geometric averaging of multiple internal control genes. Genome Biol.

[19] Wang D, Garcia-Bassets I, Benner C, Li W, Su X, Zhou Y (2011). Reprogramming transcription by distinct classes of enhancers functionally defined by eRNA. Nature.

[20] Langmead B, Trapnell C, Pop M, Salzberg SL (2009). Ultrafast and memory-efficient alignment of short DNA sequences to the human genome. Genome Biol.

[21] Robinson MD, McCarthy DJ, Smyth GK (2010). edgeR: a bioconductor package for differential expression analysis of digital gene expression data. Bioinformatics.

[22] Schmidt D, Wilson MD, Spyrou C, Brown GD, Hadfield J, Odom DT (2009). ChIP-seq: using high-throughput sequencing to discover protein–DNA interactions. Methods.

[23] Li H, Handsaker B, Wysoker A, Fennell T, Ruan J, Homer N (2009). The sequence alignment/map format and SAMtools. Bioinformatics.

[24] Zhang Y, Liu T, Meyer CA, Eeckhoute J, Johnson DS, Bernstein BE (2008). Model-based analysis of ChIP-Seq (MACS). Genome Biol.

[25] Ramirez F, Dundar F, Diehl S, Gruning BA, Manke T (2014). deepTools: a flexible platform for exploring deep-sequencing data. Nucleic Acids Res.

[26] Quinlan AR, Hall IM (2010). BEDTools: a flexible suite of utilities for comparing genomic features. Bioinformatics.

[27] Hurtado A, Holmes KA, Ross-Innes CS, Schmidt D, Carroll JS (2011). FOXA1 is a key determinant of estrogen receptor function and endocrine response. Nat Genet.

[28] Zhou Y, Zhou B, Pache L, Chang M, Khodabakhshi AH, Tanaseichuk O (2019). Metascape provides a biologist-oriented resource for the analysis of systems-level datasets. Nat Commun.

[29] Subramanian A, Tamayo P, Mootha VK, Mukherjee S, Ebert BL, Gillette MA (2005). Gene set enrichment analysis: a knowledge-based approach for interpreting genome-wide expression profiles. Proc Natl Acad Sci USA.

