# Supplementary material for: The proapoptotic gene interferon regulatory factor-1 mediates the antiproliferative outcome of paired box 2 gene and tamoxifen
Source: Oncogene. 2020 Aug 25;39(40):6300–12. doi: 10.1038/s41388-020-01435-4 (PMC7529584; doi:10.1038/s41388-020-01435-4)
Supplement: Supplementary file 1 — Supplementary Figure Legend [file 41388_2020_1435_MOESM1_ESM.docx]

**Supplementary Figures**

**Supplementary Figure 1: PAX2 and tamoxifen represses estrogen signaling.** List of enriched pathways in genes down regulated by (A)Tamoxifen, (B) PAX2 and (C) PAX2+Tamoxifen.

**Supplementary Figure 2: PAX2 and tamoxifen induce pathways related to growth arrest and cell death.** List of enriched pathways in genes up-regulated

(A)tamoxifen, (B) PAX2 and (C) PAX2+tamoxifen.

**Supplementary Figure 3: Gene set enrichment analysis (GSEA) in estrogen signaling of genes repressed in GRO-seq.** Enrichment plots and tables showing enrichment scores (ES), normalized enrichment scores (NES), and false discovery ratio (FDR) of genes repressed in GRO-seq on estrogen early (A) and estrogen late (B) signaling.

**Supplementary Figure** 4**: PAX2-HA ChIP-sequencing in MCF-7 cells.** (A and B) Venn Diagram showing the overlap for two biological replicates of PAX2-HA ChIP-sequencing (A) or PAX2-HA with tamoxifen ChIP-sequencing (B). Replicate 1(Rep1) is indicated in blue and replicate 2 (Rep2) in red. Pearson correlation analysis to determine the confidence in the peaks called in any of the biological replicates for each treatment. We pulled all the peaks for each biological replicate and we obtained 84,730 PAX2-HA peaks in (C) non-tamoxifen treated cells and 90,557 PAX2-HA peaks in (D) tamoxifen treated cells. The analysis reveals a very high correlation for both biological replicates. (E). Chromatin from MCF-7 cells growth with Doxocycline 50mg/ml (Dox) or control (Veh) was isolated and chromatin was pulled down with HA antibody. Then, ChIP-PCR of genes with PAX2 binding (FGF18, ARHGEF4 and SCNN1G) was tested. The average of HA-PAX2 interaction of two biological replicates is shown.

**Supplementary Figure 5: PAX2-HA binding is modulated by tamoxifen.** (A) Venn Diagram of PAX2-HA ChIP-sequencing data of MCF7-PAX2-HA induced cells with doxycycline (DOX; 50 ng/ml) for 16h and control or tamoxifen treated for 6h. (B) Heatmap showing the signal intensity of PAX-HA binding to the chromatin in MCF7-PAX2-HA cells non-induced (Veh), induced with doxycycline at 50 ng/ml (control) or doxycycline induced and treated with tamoxifen for 6h (DoxTam). (C) Heatmap showing the signal intensity of PAX-HA binding to the transcription start sites (TSS) of PAX2, PAX2-tamoxifen or tamoxifen regulated genes in MCF7-PAX2-HA). The fraction of genes (indicated in percentage) with PAX2-HA binding at TSS of each cluster of genes is indicated. The top heatmap indicates genes up-regulated and the bottom heatmap indicates the genes down-regulated. (D) mRNA level and PAX2 binding on a few selected PAX2 induced genes in TNF or p53 pathways when treated without or with tamoxifen.

**Supplementary Figure 6: complement of Supplementary figure 5.** (A) Venn Diagram showing the scheme of the grouping of differential expressed genes identified in GRO-seq and regulated by tamoxifen (Tam), PAX2 overexpression with 50mg/ml of Doxocycline (Dox) or with the combination of tamoxifen (DoxTam). Seven group of genes for each treatment was identified. (B) The number of genes for each group of up-regulated down-regulated is indicated. (C) PAX2 binding at non-PAX2 regulated genes. The binding was analyzed at PAX-HA binding to the chromatin in MCF7-PAX2-HA cells induced with Doxocycline at 50 ng/ml (control) or doxocycline induced and treated with tamoxifen for 6h (DoxTam). The results indicate that PAX2-HA do not show enrichment at chromatin regions (TSS) of genes non-PAX2 regulated (n=500).

**Supplementary Figure 7: geNORM analysis of 8 reference genes tested in qPCR experiment.** (A) stability score of each reference gene (B) recommendation of the number of reference genes to be used. (C) qPCR analysis of a few PAX2 regulated genes with normalization to UBC and RPL13A.

**Supplementary Figure 8: Tamoxifen shows an additive effect in expression on PAX2 regulated genes.** (A) Boxplot showing the log2 fold change of common down-regulated genes shared by tamoxifen treatment and PAX2 overexpression treatment in all 3 treatments, numbers of genes are indicated. (B) Boxplot showing the log2 fold change of common down-regulated genes shared by tamoxifen treatment and PAX2 overexpression treatment that enriched in estrogen signaling in all 3 treatments, number of genes is indicated. (C) Real-time PCR of selected genes repressed by both tamoxifen and PAX2 when treated with vehicle (Veh), tamoxifen (Tam), doxycycline (Dox), and the combination of tamoxifen and doxycycline (DoxTam). Relative mRNA level is normalized against UBC and RPL13A, then to the average of the Veh. The data is represented as the mean of independent replicates ± s.d (n = 3, *, p<0.05, **, p<0.01). (D-E) Boxplot showing the log2 fold change of PAX2 up-regulated genes that enriched in TNF alpha (D) and p53 (E) pathways in different treatments. Red dots indicate genes with gained PAX2 binding upon tamoxifen treatment as described in Figure 2D. (F) Real-time PCR of selected PAX2 up-regulated genes that enriched in TNF alpha and/or p53 pathways. (G) IRF1 and p53 protein level with PAX2 overexpression and tamoxifen. (H) qPCR experiment showing p53 mRNA level with different treatments.

**Supplementary Figure 9: PAX2 induces transcription of eRNAs.** (A, B, C) Venn Diagram showing the scheme of the grouping of differential expressed eRNAs identified in GRO-seq and regulated by tamoxifen (Tam), PAX2 overexpression with 50mg/ml of Doxocycline (Dox) or with the combination of tamoxifen (DoxTam). Seven group of genes for each treatment was identified. The number of genes for each group of up-regulated down-regulated is indicated. Quantification of PAX2 binding at (D) PAX2-Tamoxifen gained sites at eRNAS nearby of PAX2 up-regulated genes and (E) PAX2- gained sites at eRNAS nearby of PAX2 up-regulated genes.

**Supplementary Figure 10: Complement of Figure 4.** Real-time PCR analysis of genes regulated by PAX2 with IRF1 knocking down. MCF-7-PAX2 cells were transfected with non-targeting siRNA or siIRF1 followed by doxycycline treatment to induce PAX2 expression. mRNA level of a few PAX2 up-regulated genes were tested with real-time PCR.

**Supplementary Figure 11: Complement of Figure 5**. Real-time images showing cell confluence and annexin V signal. MCF-7-PAX2 cells were transfected with non-targeting siRNA or siIRF1 followed by doxycycline (Dox), tamoxifen (Tam), or the combination (DoxTam) treatments. Cell confluence is shown as phase contrast, and annexin V signal is shown as red fluorescent signal.

**Supplementary Figure 12:** PAX2 inhibits cell growth and induces apoptosis together with tamoxifen in ZR-75-1 cells. (A) Western blotting showing PAX2 over-expression in ZR-75-1. (B) Cell proliferation assay showing the effect of PAX2 on cell proliferation. (C) Real-time Annexin V assay with PAX2 over-expression and tamoxifen treatment.
