# Supplementary material for: The proapoptotic gene interferon regulatory factor-1 mediates the antiproliferative outcome of paired box 2 gene and tamoxifen
Source: Oncogene. 2020 Aug 25;39(40):6300–12. doi: 10.1038/s41388-020-01435-4 (PMC7529584; doi:10.1038/s41388-020-01435-4)

Supplementary Figure 1

A

Tam down-regulated

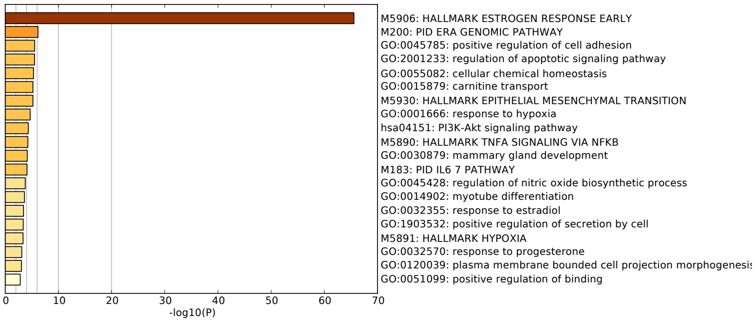

B

PAX2 down-regulated

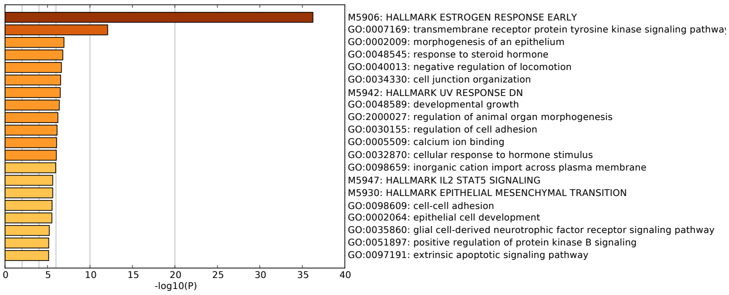

C

PAX2 Tam down-regulated

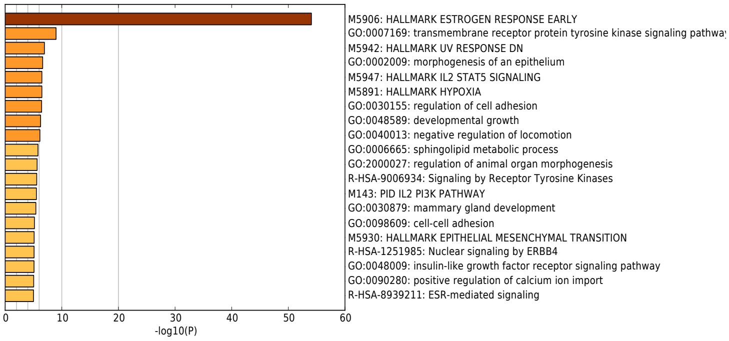

Supplementary Figure 2

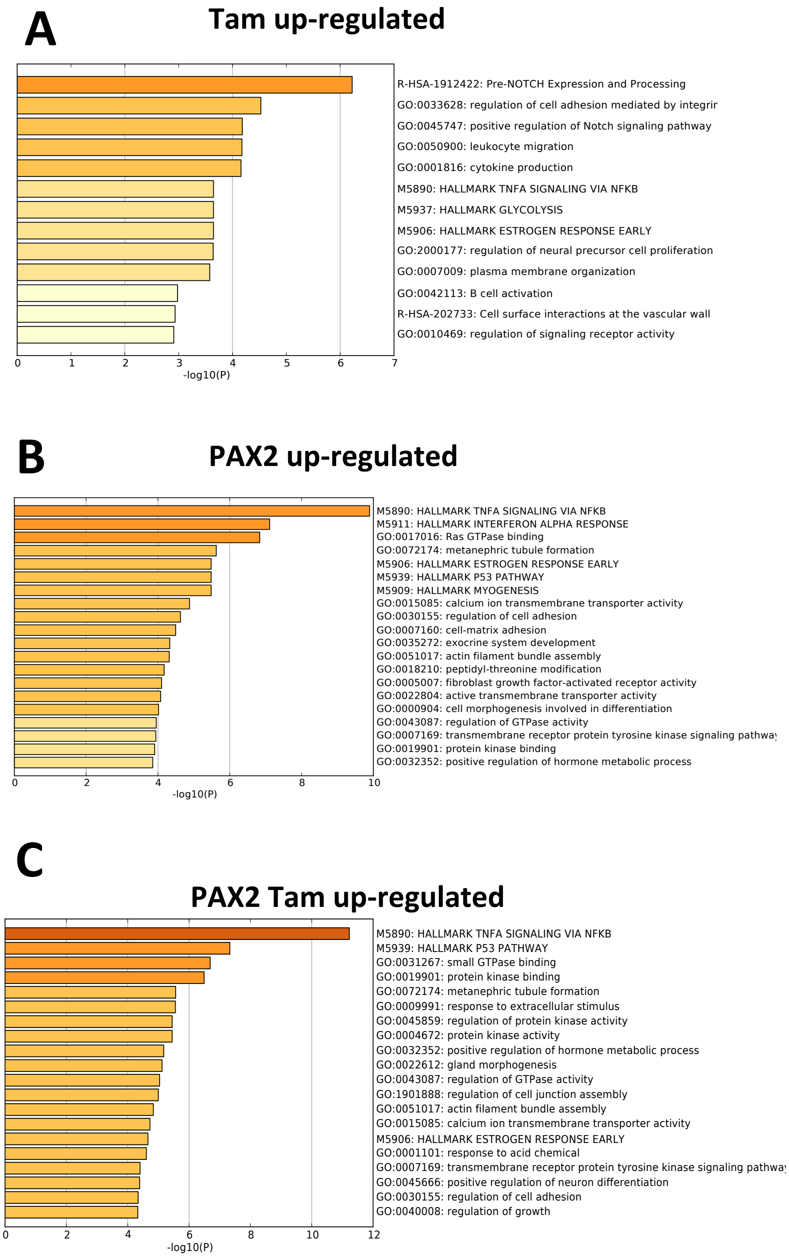

Supplementary Figure 3

A Tam Dox DoxTam

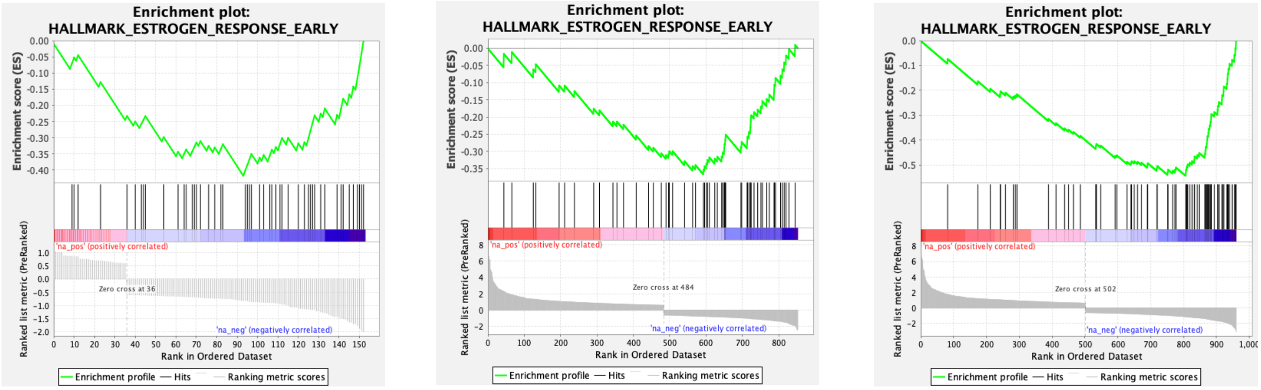

Estrogen response early	ES	NES	FDR
Tam	-0.42	-1.97	0.002
Dox	-0.37	-2.37	0.002
DoxTam	-0.54	-3.53	0.000

B Tam Dox DoxTam

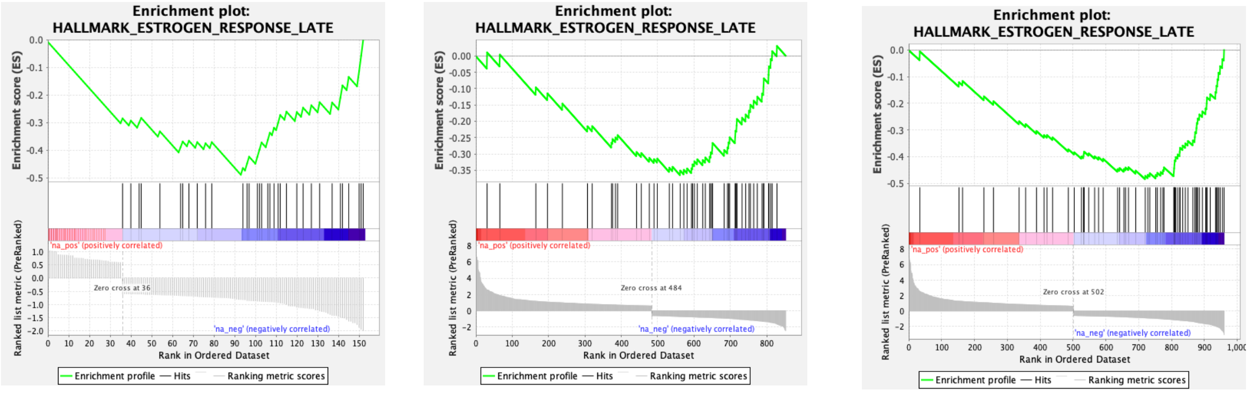

Estrogen response late	ES	NES	FDR
Tam	-0.49	-2.15	0.001
Dox	-0.36	-2.3	0.002
DoxTam	-0.48	-3.02	0.000

Supplementary Figure 4

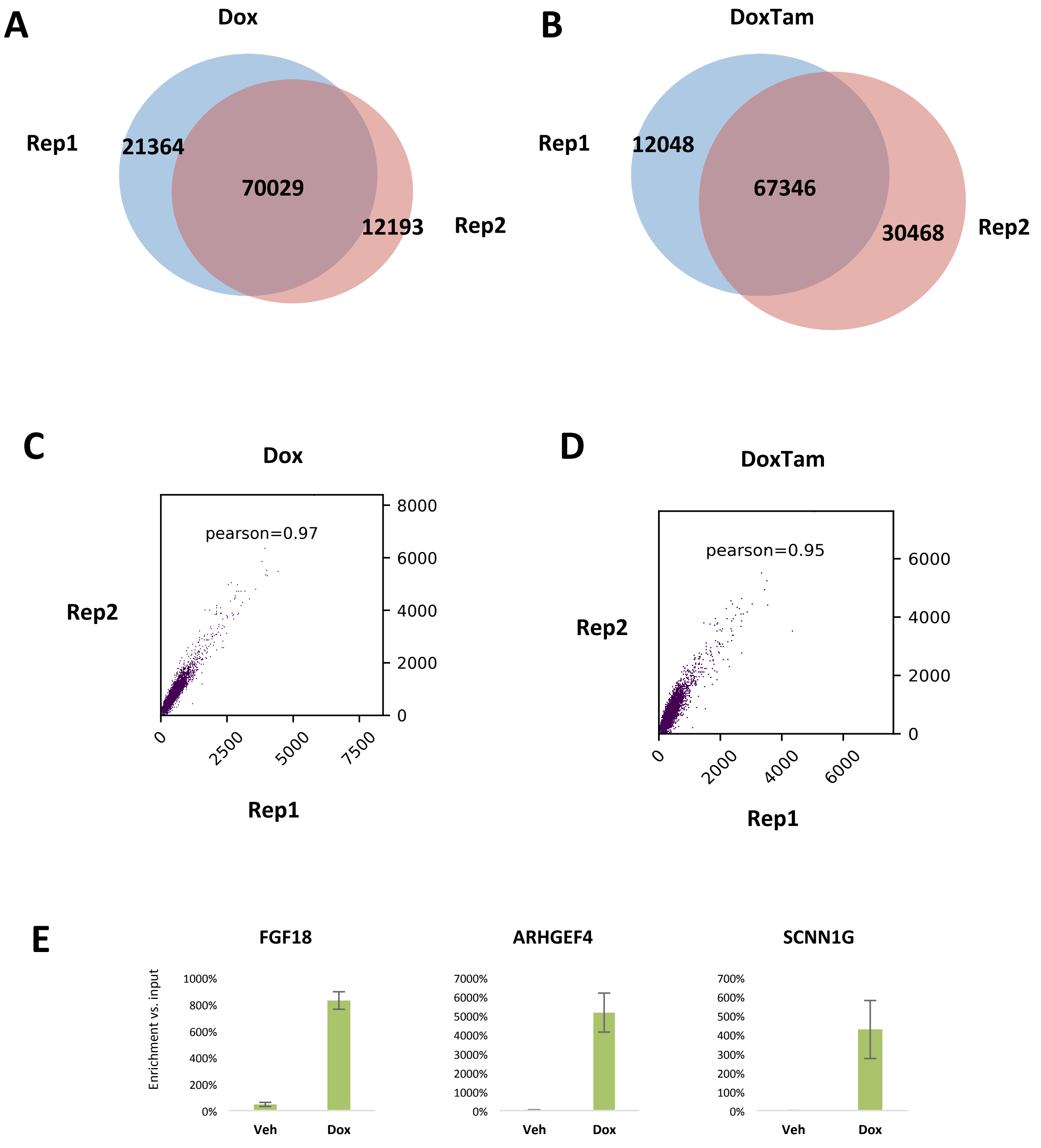

Supplementary Figure 5

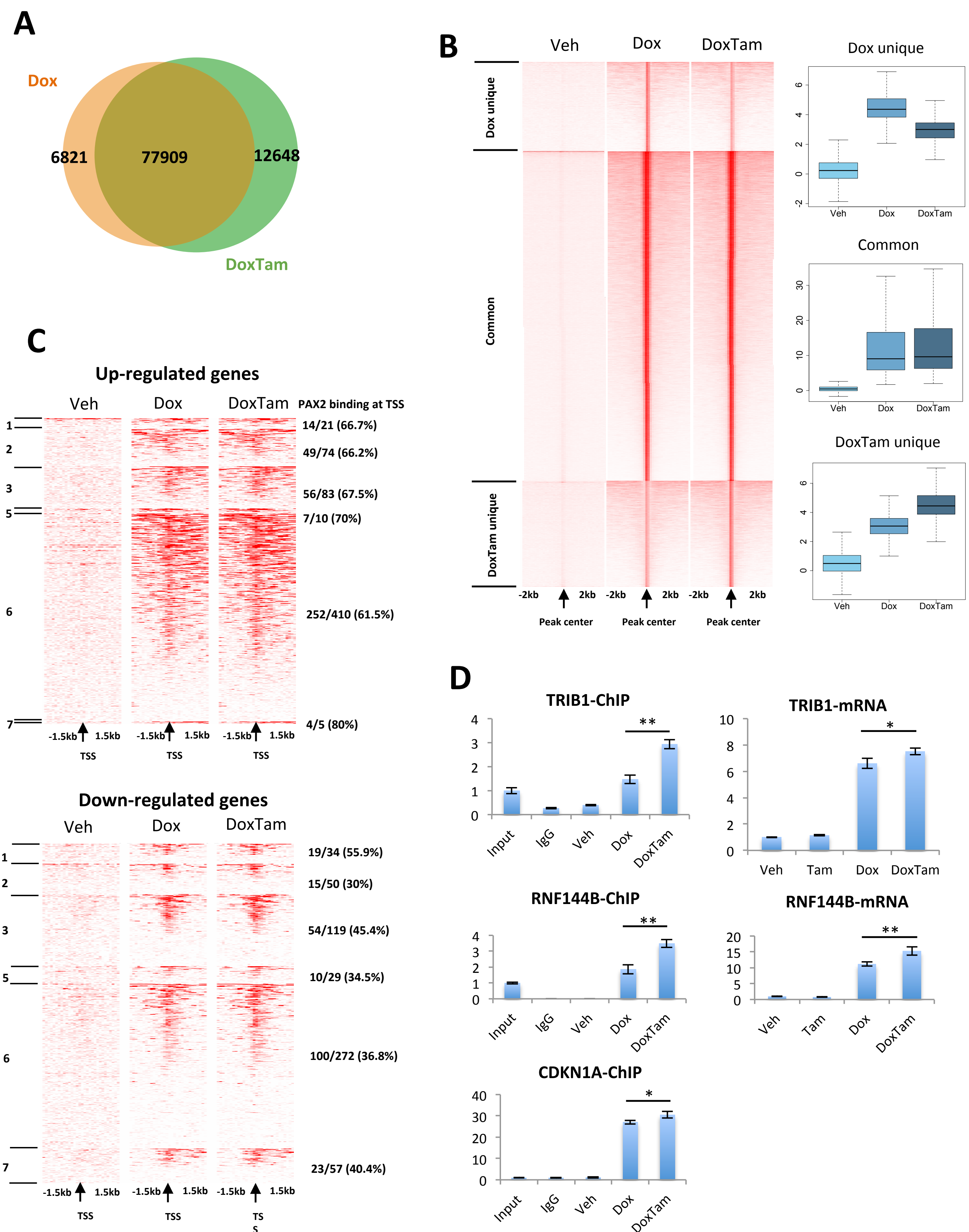

Supplementary Figure 6

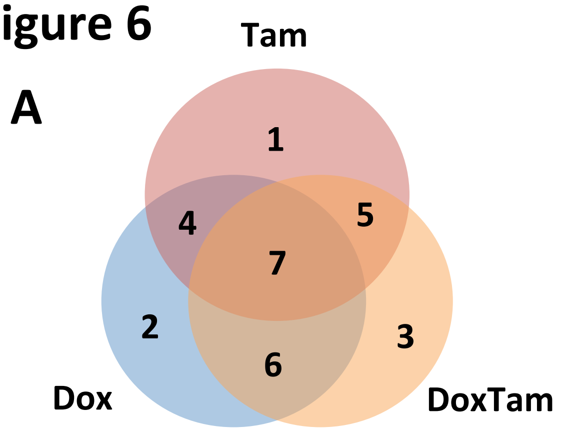

B

Group	No. of up reg genes	No. of down reg genes
1	21	34
2	74	50
3	83	119
4	0	0
5	10	29
6	410	272
7	5	57

C PAX2 binding on TSS of 500 non-reg genes

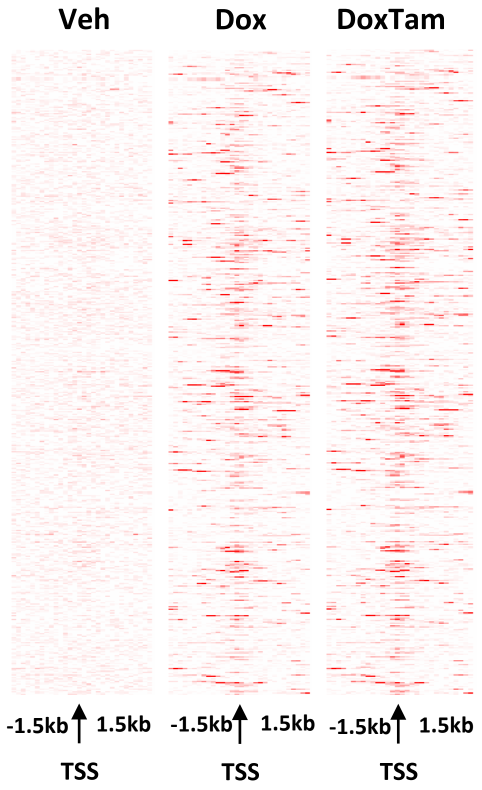

Supplementary Figure 7

A geNORM analysis of 8 reference genes in treatments of GRO-seq

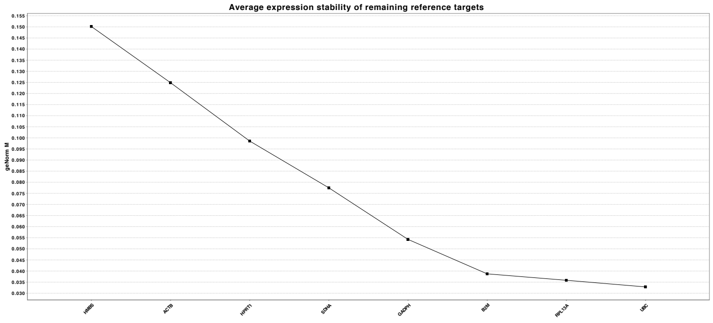

B

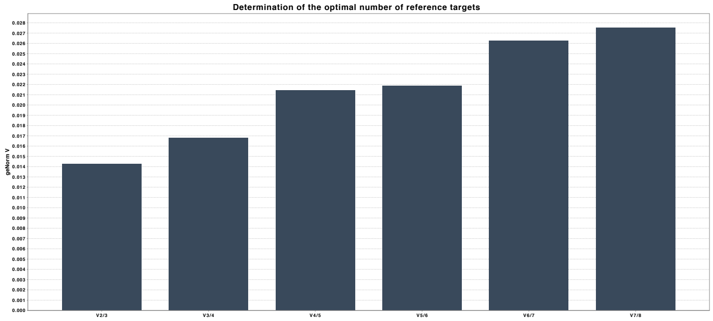

Optimal reference target selection
The optimal number of reference targets in this experimental situation is 2 (geNorm V < 0.15 when comparing a normalization factor based on the 2 or 3 most stable targets). As such, the optimal normalization factor can be calculated as the geometric mean of reference targets RPL13A and UBC.

C

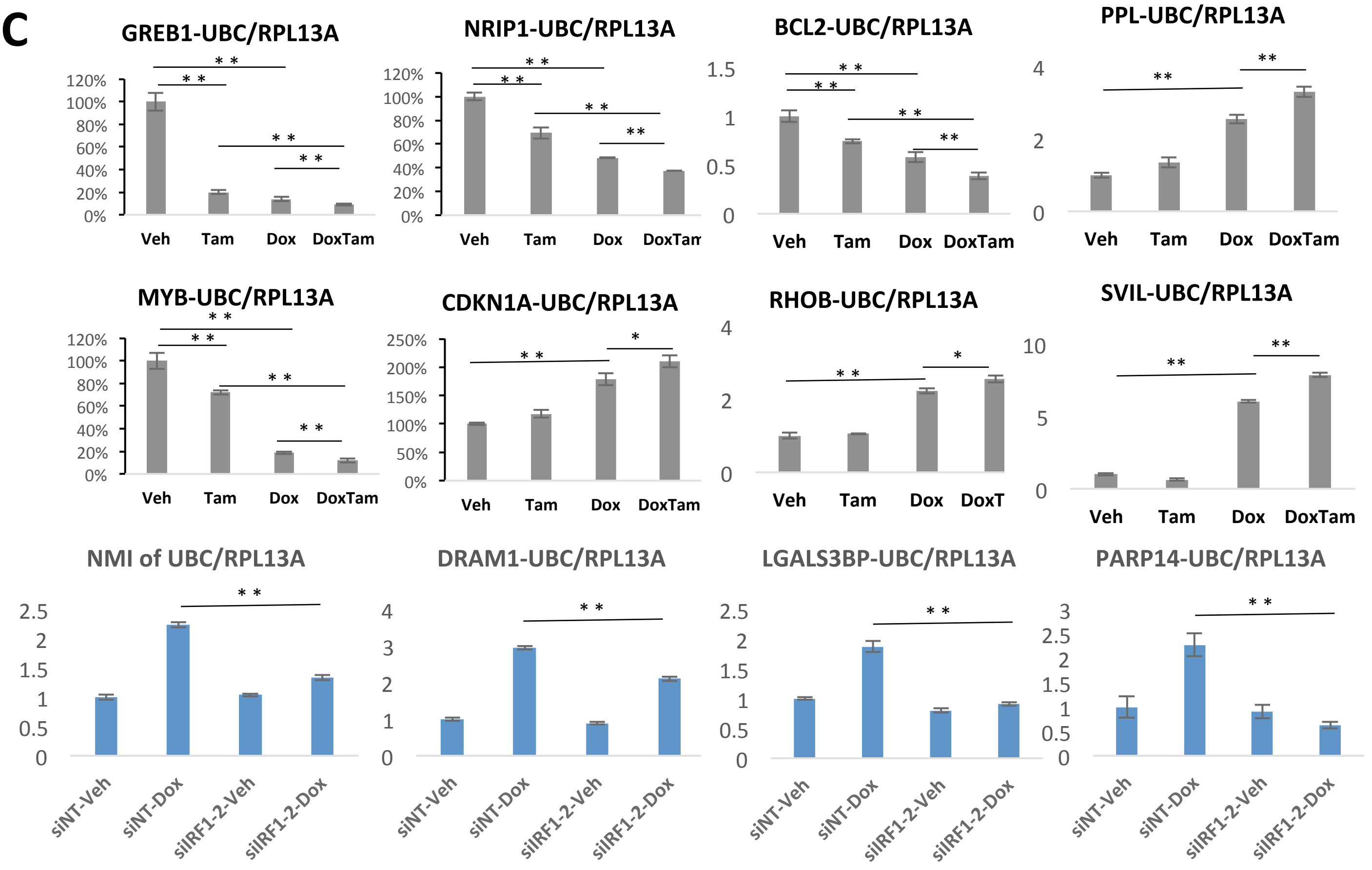

Supplementary Figure 8

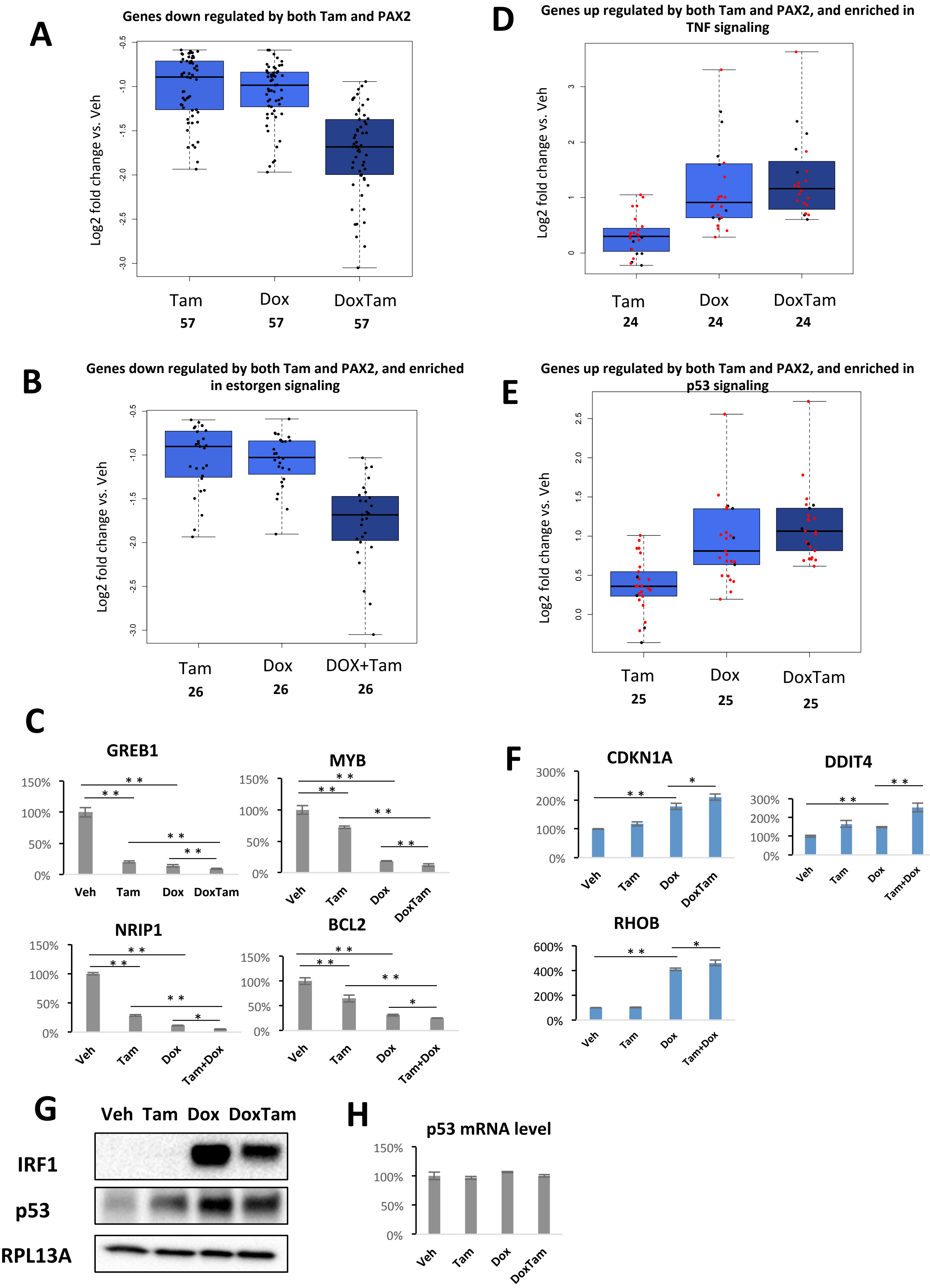

Supplementary Figure 9

A

Treatments	Up	Down
Tam	9	68
PAX2	173	245
Tam + PAX2	209	298

B

Up regulated

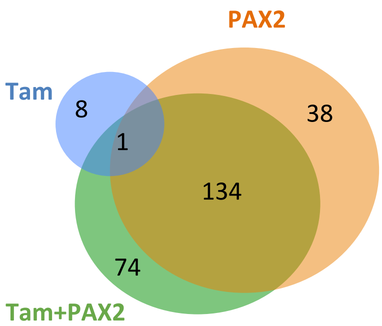

C

Down regulated

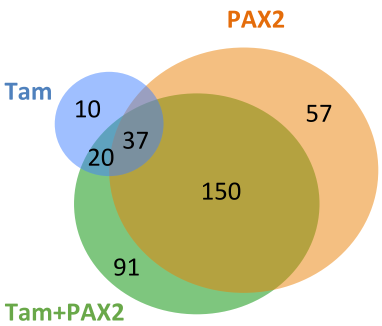

D

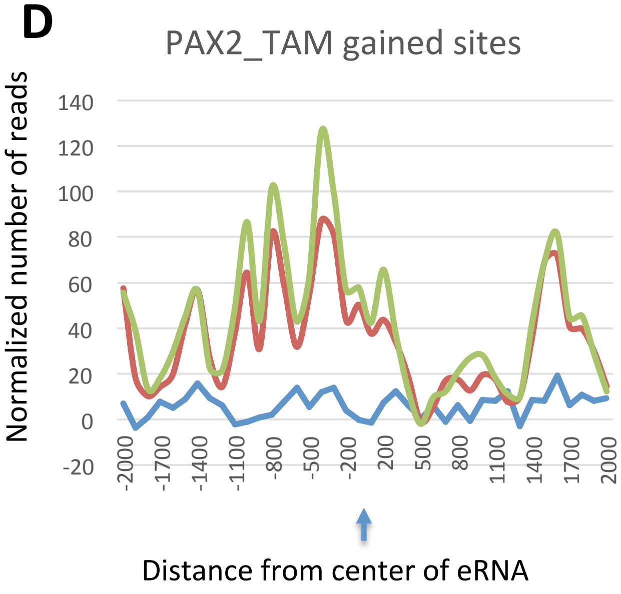

E

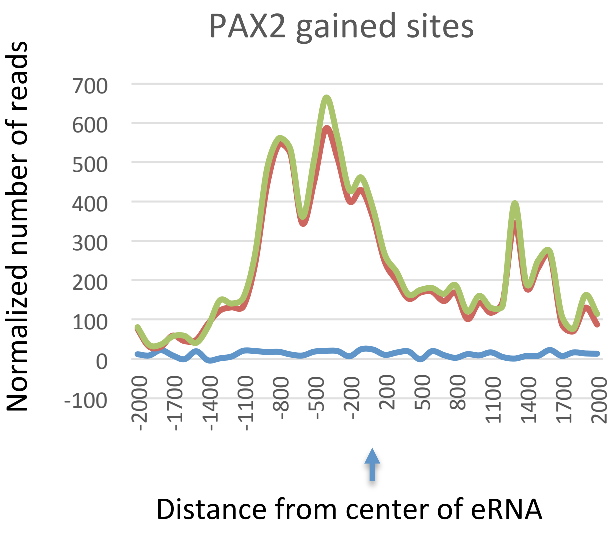

Veh Dox DoxTam

Supplementary Figure 10

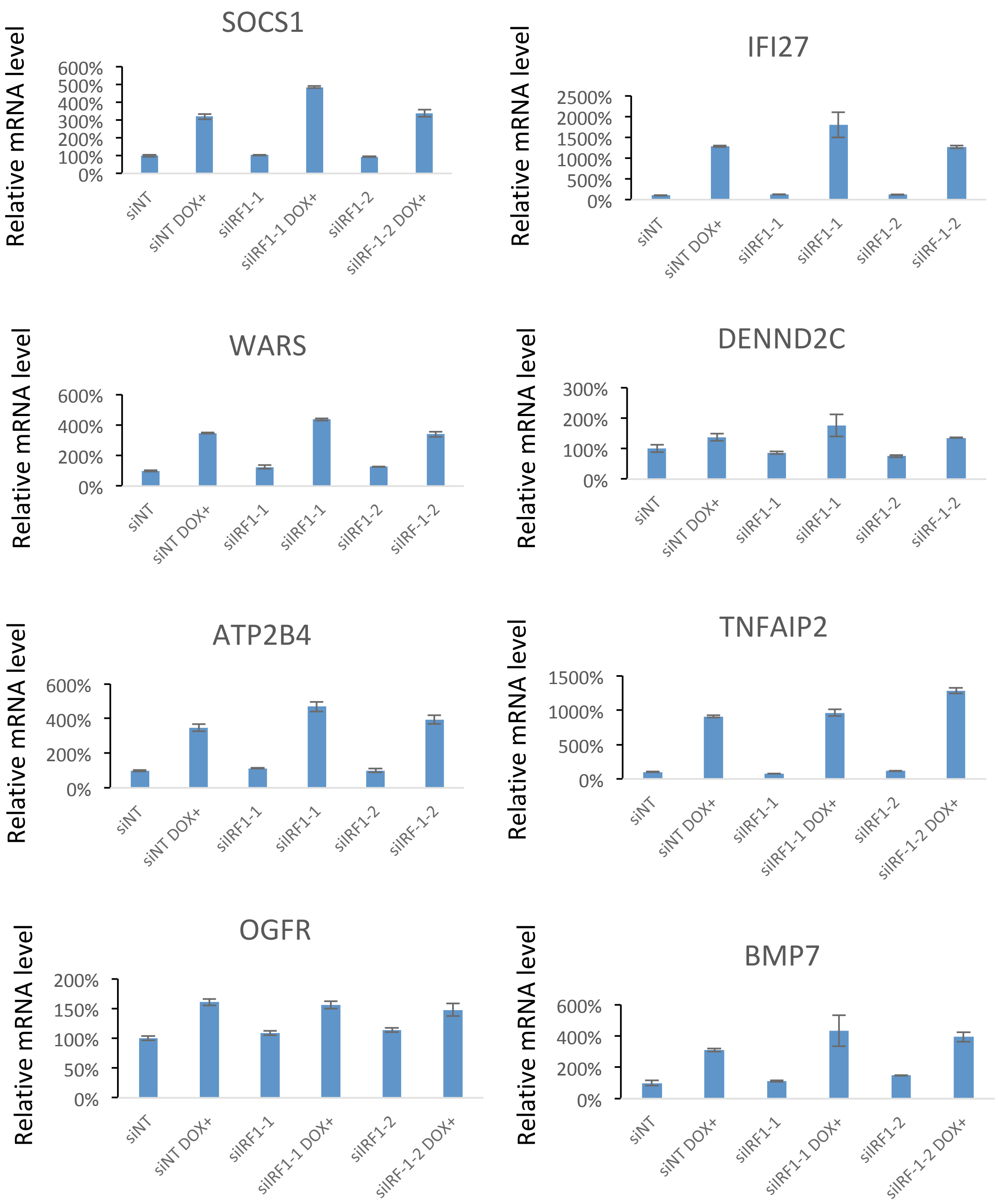

Supplementary Figure 11

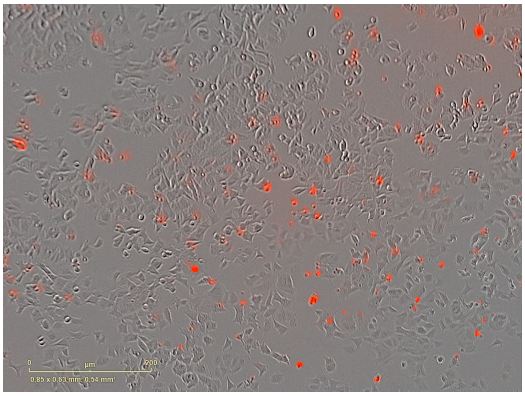

siNT-Veh

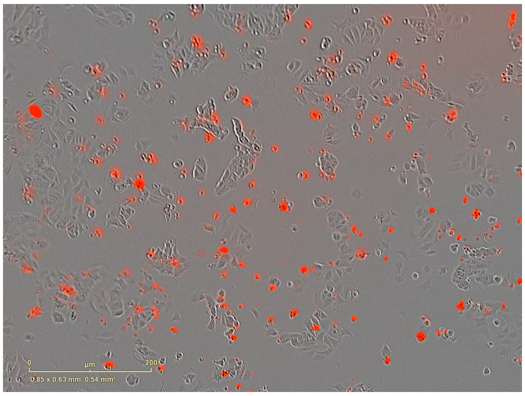

siNT-Dox

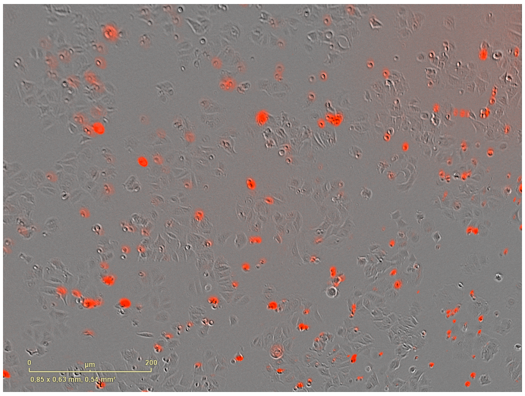

siNT-Tam

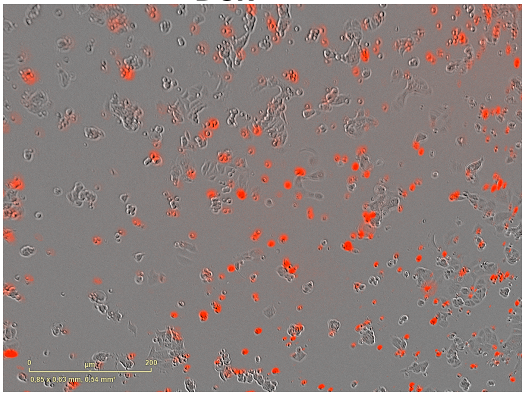

siNT-Dox+Tam

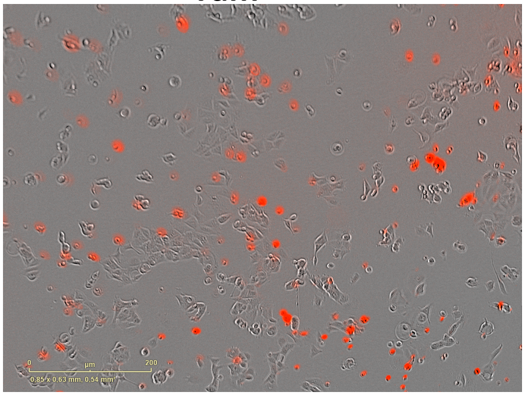

siIRF1-1-Dox+Tam

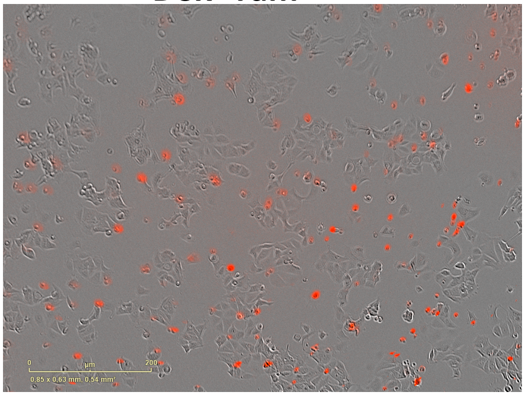

siIRF1-2-Dox+Tam

Supplementary Figure 12

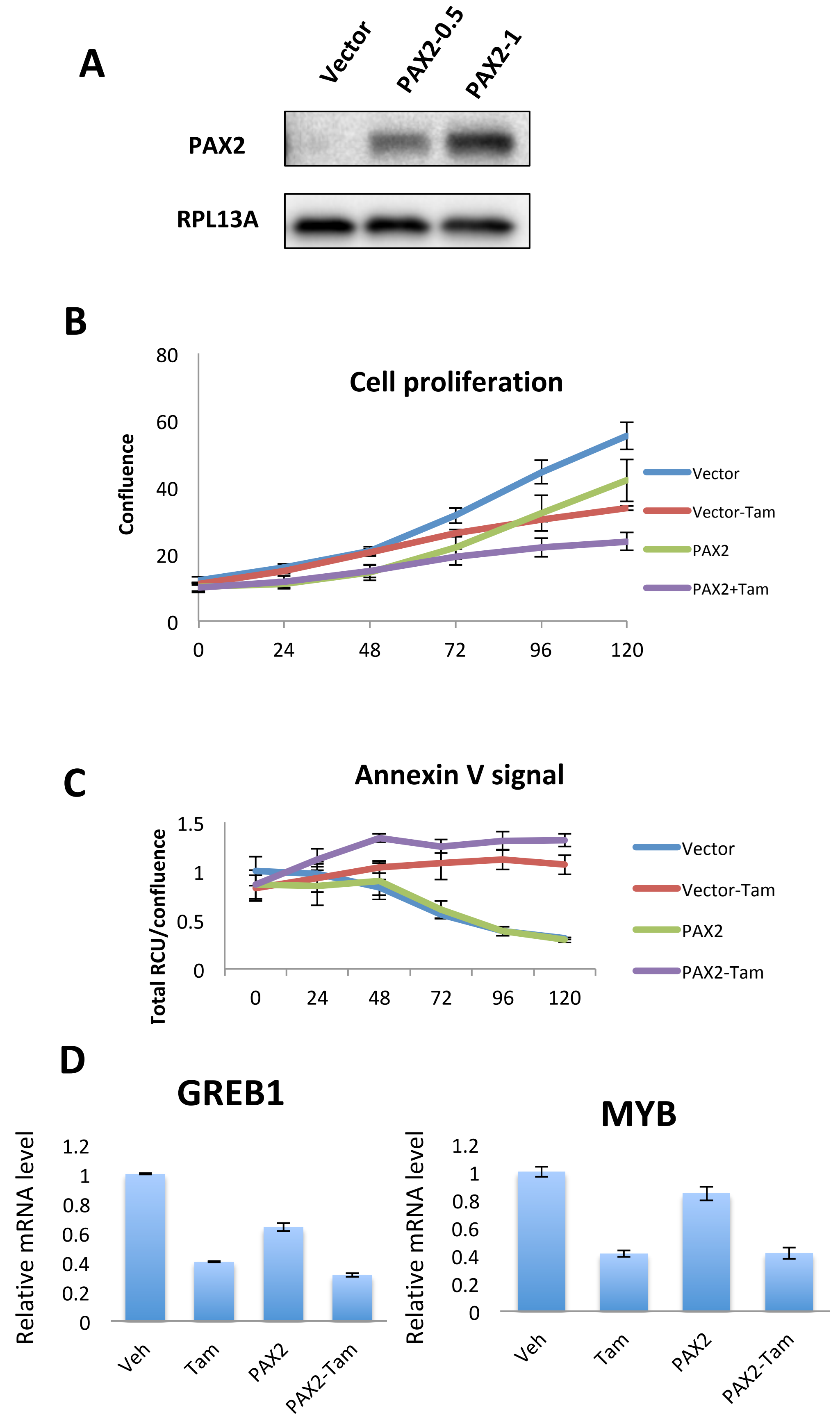

Supplement: Supplementary file 2 — Supplementary figures [file 41388_2020_1435_MOESM2_ESM.pdf]
