# Supplementary material for: The proapoptotic gene interferon regulatory factor-1 mediates the antiproliferative outcome of paired box 2 gene and tamoxifen
Source: Oncogene. 2020 Aug 25;39(40):6300–12. doi: 10.1038/s41388-020-01435-4 (PMC7529584; doi:10.1038/s41388-020-01435-4)
Supplement: Supplementary file 3 — Supplementary materials [file 41388_2020_1435_MOESM3_ESM.docx]

GRO-seq analysis pipeline:

Differential gene expression analysis:

1. Reads are trimmed from 3’ end so that poly-A tail is removed with homerTools (Homer v4.6), min read is kept at 25bp

homerTools trim -3 AAAAAAAAA -min 25

1. FASTX Toolkit (0.0013) is used for Quality filtering (min. 97% of bases should have quality score of 10)

fastq_quality_filter -v -q 10 -p 97 -i input file -o output file

1. Aligning with hg19 genome with Bowtie (1.1.2)

bowtie hg19 -q -v 2 -m 3 -k 1

1. Make tag directories with Homer

makeTagDirectory <directory> <alignment file 1>

1. Count reads for each gene with Homer

analyzeRepeats.pl rna hg19 -noadj -condenseGenes -d PAX2_C_1/ PAX2_C_2/ PAX2_TAM_1/ PAX2_TAM_2/ PAX2_DOX_1/ PAX2_DOX_2/ PAX2_DOX_TAM_1/ PAX2_DOX_TAM_2/ > analyzeRepeat_PAX2_noadj.txt

1. For actual differential expression analysis you input the analyzeRNA output file (noadj) into getDiffExpression.pl (DeSeq2 invoked by Homer)

getDiffExpression.pl analyzeRepeat_PAX2_noadj.txt Veh Veh TAM TAM DOX DOX DOX+TAM DOX+TAM -batch 1 2 1 2 1 2 1 2 > getDiffExpression_PAX2_batch.txt

Enhancer detection and differential analysis:

1. Tags from all samples are pooled with Homer:

makeTagDirectory PAX2_Comb -d PAX2_C_1/ PAX2_C_2/ PAX2_DOX_1/ PAX2_DOX_2/ PAX2_DOX_TAM_1/ PAX2_DOX_TAM_2/ PAX2_TAM_1/ PAX2_TAM_2/

1. findPeaks command was used to find transcripts from GRO-seq data

findPeaks PAX2_Comb/ -style groseq -minBodySize 300 > findPeaks_PAX2_Comb_min300.txt

1. Sorting for intergenic transcripts with getDistalPeaks.pl command

getDistalPeaks.pl findPeaks_PAX2_Comb_min300.txt hg19 -intergenic -noTTS > getDistalPeaks_PAX2_findPeaksMin300_intergenic_noTTS.txt

1. All Refseq sequences removed with getDistalPeaks.pl command

getDistalPeaks.pl getDistalPeaks_PAX2_findPeaksMin300_intergenic_noTTS.txt hg19 -intergenic -noTTS -gtf hg19_RefSeq_genes_16_5_2016_GTF.txt > getDistalPeaks_PAX2_findPeaksMin300_intergenic_noTTS_RefSeqExcluded.txt

1. Counting the tags from enhancer regions

analyzeRepeats.pl getDistalPeaks_PAX2_findPeaksMin300_intergenic_noTTS_RefSeqExcluded.txt hg19 -d PAX2_C_1/ PAX2_C_2/ PAX2_TAM_1/ PAX2_TAM_2/ PAX2_DOX_1/ PAX2_DOX_2/ PAX2_DOX_TAM_1/ PAX2_DOX_TAM_2/ -noadj > analyzeRepeats_PAX2_findPeaksMin300_intergenic_noTTS_RefSeqExcluded _noadj.txt

1. differentially expressed intergenic transcripts were called finally

getDiffExpression.pl analyzeRepeats_PAX2_findPeaksMin300_intergenic_noTTS_RefSeqExcluded_noadj.txt Veh Veh Tam Tam Dox Dox DoxTam DoxTam -batch 1 2 1 2 1 2 1 2 > getDiffExpression_PAX2_findPeaksMin300_intergenic_noTTS_RefSeqExcluded.txt

ChIP-seq analysis pipeline:

1. First data are aligned to hg19 with Bowtie

bowtie hg19 -q -v 2 -m 3 -k 1

1. Sam files are converted to bam files and bam files are sorted with samtools(1.3.1)

samtools view -bS/ samtools sort

1. Duplicate reads are removed with samtools

samtools rmdup -s

1. Bam files are indexed

samtools index

1. Peaks are called with MACS2 (2.1.1) with q value 0.01

callpeak -t sample.bam -c input.bam -g hs -n output -q 0.01
